# Supplementary material for: Conservation of epigenetic regulation by the MLL3/4 tumour suppressor in planarian pluripotent stem cells
Source: Nat Commun. 2018 Sep 7;9:3633. doi: 10.1038/s41467-018-06092-6 (PMC6128892; doi:10.1038/s41467-018-06092-6)
Supplement: Supplementary file 1 — Supplementary Information [file 41467_2018_6092_MOESM1_ESM.pdf]

Conservation of epigenetic regulation by the MLL3/4 tumour suppressor in planarian pluripotent stem cells

Mihaylova et al.

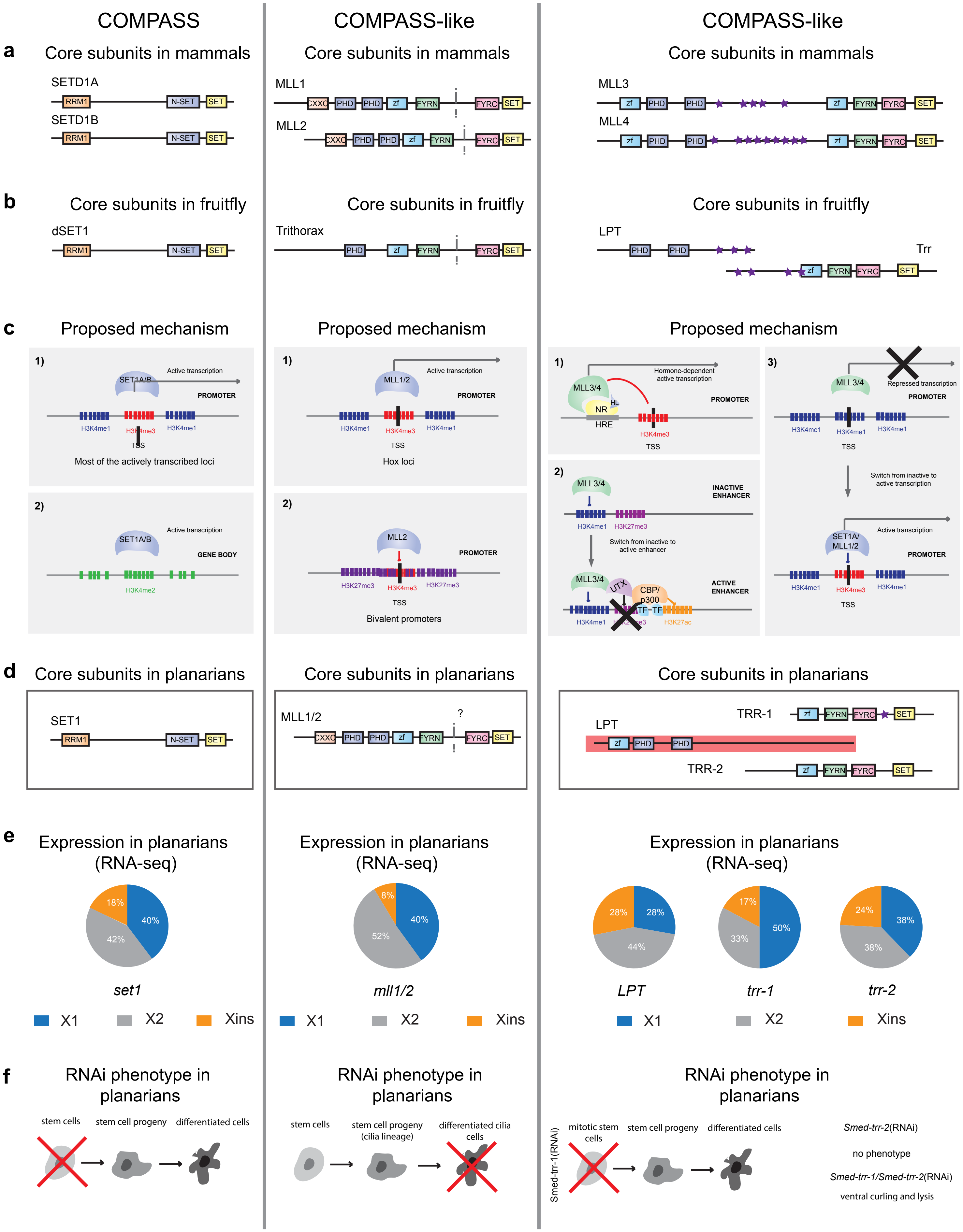

COMPASS
COMPASS-like
COMPASS-like
a
Core subunits in mammals
SETD1A
RRM1
N-SET
SET
SETD1B
MLL1
CXXC
PHD
zf
FYRN
FYRC
MLL2
MLL3
MLL4
b
Core subunits in fruitfly
dSET1
Trithorax
LPT
Trr
c
Proposed mechanism
1)
SET1A/B
Active transcription
PROMOTER
H3K4me1
H3K4me3
TSS
Most of the actively transcribed loci
2)
GENE BODY
H3K4me2
MLL1/2
Hox loci
H3K27me3
Bivalent promoters
MLL3/4
Hormone-dependent active transcription
NR
HL
HRE
INACTIVE ENHANCER
Switch from inactive to active enhancer
UTX
CBP/ p300
TF
ACTIVE ENHANCER
H3K27ac
3)
Repressed transcription
Switch from inactive to active transcription
SET1A/ MLL1/2
d
Core subunits in planarians
SET1
?
TRR-1
TRR-2
e
Expression in planarians (RNA-seq)
18%
40%
42%
set1
8%
52%
mll1/2
28%
28%
44%
LPT
17%
33%
50%
trr-1
24%
38%
38%
trr-2
X1
X2
Xins
f
RNAi phenotype in planarians
stem cells
stem cell progeny
differentiated cells
stem cell progeny (cilia lineage)
differentiated cilia cells
mitotic stem cells
Smed-trr-1(RNAi)
Smed-trr-2(RNAi)
no phenotype
Smed-trr-1/Smed-trr-2(RNAi)
ventral curling and lysis

Supplementary Figure 1. Structure and function of COMPASS and COMPASS-like core proteins.

a Schematics of the core subunits of the COMPASS and the two COMPASS-like complexes in mammals are presented with coloured boxes corresponding to different protein domains – RRM1 (RNA-recognition motif), N-SET, SET, CXXC (zinc finger), PHD (Plant Homeodomain fingers), zf (PHD-like zinc finger), FYRN (Phenylalanine/Tyrosine rich N-terminus domain), FYRC (Phenylalanine/Tyrosine rich C-terminus domain), purple stars signifying nuclear receptor recognition motifs. Dashed vertical line represents proteolytic cleavage. **b** As in (**a**), but in fruitfly. **c** Proposed mechanisms of action of each core complex subunit. COMPASS complex – 1) performing H3K4 trimethylation on TSS of most actively transcribed genes and 2) depositing H3K4me2 on the gene bodies of actively transcribed genes. MLL1/2/Trithorax COMPASS-like complex – 1) a role in transcriptional activation of Hox genes via trimethylating H3K4 on TSS of their promoters and 2) MLL2 is involved in trimethylation of H3K4 on TSS of bivalent promoters. MLL3/4/LPT/Trr – 1) role in hormone-dependent transcription – when the Nuclear Receptor protein (NR) is bound to the DNA Hormone Response Element (HRE) upon Hormone Ligand (HL) detection, MLL3/4/LPT/Trr complex binds the nuclear receptor and serves as its co-activator via trimethylating H3K4 and promoting active transcription on selected loci; 2) a switch between inactive and active enhancer states where MLL3/4/LPT/Trr complex deposits H3K4me1 on both active and inactive enhancers; upon UTX recruitment, it demethylates H3K27me3 and allows for CBP/p300 to acetylate H3K27 and activate the enhancer; 3) a switch between active and inactive promoters - MLL3/4/LPT/Trr complex bound to TSS deposits H3K4me1 on the TSS and around it, leads to repressed transcription of the gene; when H3K4me1 is depleted from the TSS and another complex performs trimethylation of H3K4 on TSS, this is correlated with activated transcription. **d** Schematic representation of planarian COMPASS and COMPASS-like core subunits. SMED-LPT (in red) is characterized in the present study. **e** Planarian COMPASS and COMPASS-like core subunits' expression in the three populations of cells sortable by fluorescence-activated cell sorting (FACS) (X1=G2/M stem cells, X2=G1 stem cells and stem cell progeny, X ins=differentiated cells) according to RNA-seq data. **f** Known defects after RNAi-mediated knockdown of core COMPASS and COMPASS-like subunits in planarians.

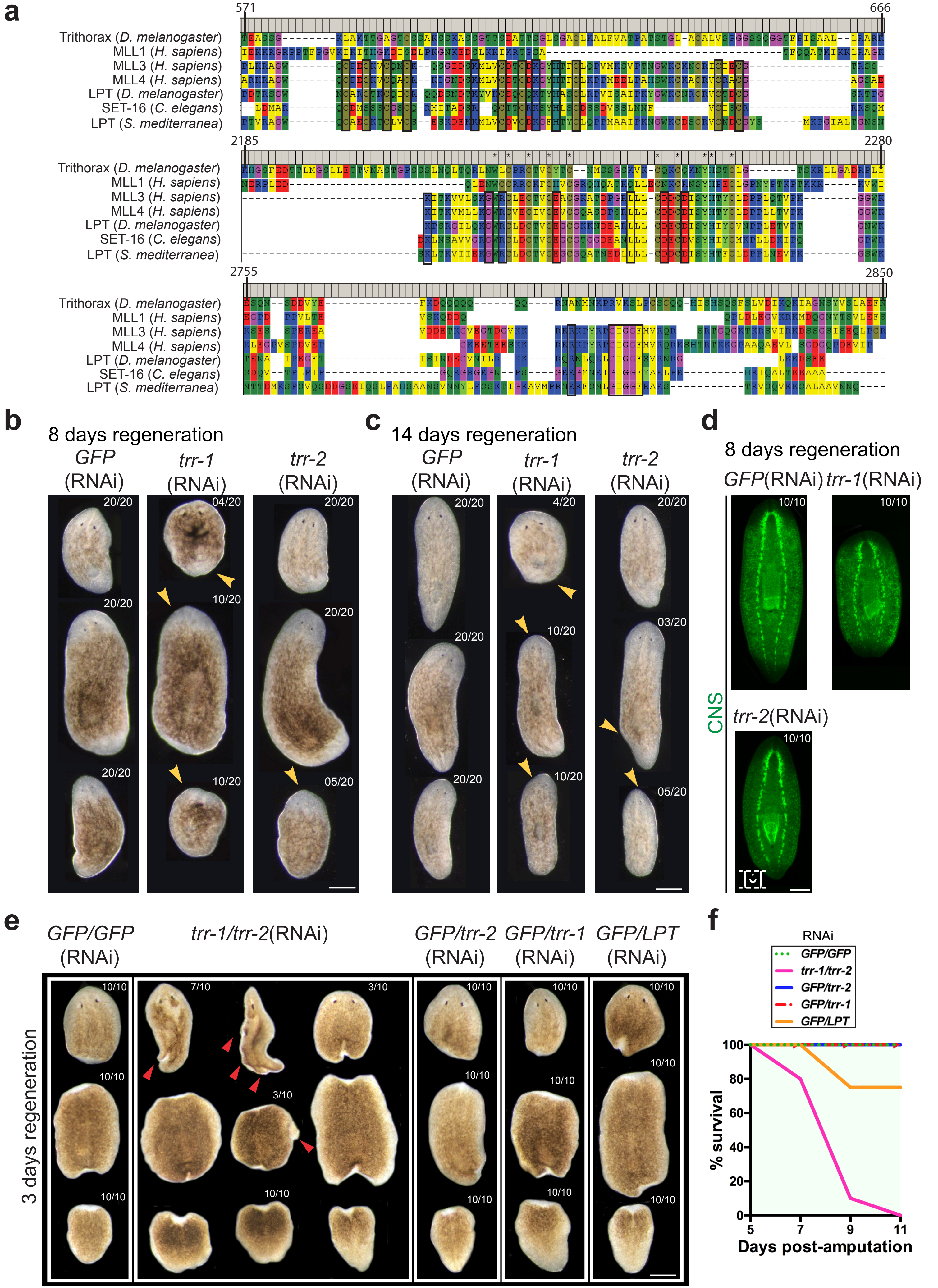
a
571
666
Trithorax (D. melanogaster)
MLL1 (H. sapiens)
MLL3 (H. sapiens)
MLL4 (H. sapiens)
LPT (D. melanogaster)
SET-16 (C. elegans)
LPT (S. mediterranea)
2185
2280
2755
2850
b
8 days regeneration
GFP (RNAi)
trr-1 (RNAi)
trr-2 (RNAi)
20/20
04/20
10/20
10/20
05/20
c
14 days regeneration
4/20
03/20
d
8 days regeneration
GFP(RNAi)
trr-1(RNAi)
trr-2(RNAi)
CNS
10/10
e
GFP/GFP (RNAi)
trr-1/trr-2(RNAi)
GFP/trr-2 (RNAi)
GFP/trr-1 (RNAi)
GFP/LPT (RNAi)
3 days regeneration
7/10
3/10
f
RNAi
GFP/GFP
trr-1/trr-2
GFP/trr-2
GFP/trr-1
GFP/LPT
% survival
Days post-amputation
0
20
40
60
80
100
5
7
9
11

Supplementary Figure 2. Planarian *Mll3/4* genes are expressed in neoblasts and neoblast progeny and colocalise with each other.

a Protein alignment of conserved regions of COMPASS-like families' core proteins. Asterisks indicate complete conservation in all sequences, while black boxes are drawn around areas of conservation specific to the MLL3/4/Trithorax-related family. Colours represent similarity of amino acids. The image was produced using MEGA.5.2 software. **b** Bright field images of head, middle and tail pieces following *trr-1*(RNAi), *trr-2*(RNAi) or control *GFP*(RNAi) at day 8 of regeneration. Yellow arrows point towards the regenerative defects – smaller blastema, delayed eye formation or posterior bloating. **c** Head, middle and tail pieces following *trr-1*(RNAi), *trr-2*(RNAi) or control *GFP*(RNAi) at day 14 of regeneration. **d** Central nervous system (CNS) maintenance and recovery at 8 days of middle piece regeneration, as labeled by CNS-specific anti-SYNORF-1 antibody, following *trr-1*(RNAi) or *trr-2*(RNAi). **e** Bright field images of head, middle and tail pieces at 3 days of regeneration following *GFP/GFP*(RNAi), *trr-1/trr-2*(RNAi), *GFP/trr-2*(RNAi), *GFP/trr-1*(RNAi) and *GFP/LPT*(RNAi). Red arrows point towards outgrowths. **f** Survival curve of middle regenerating pieces in different RNAi conditions. Green coloured dotted line is *GFP/GFP*(RNAi), magenta coloured line is *trr-1/trr-2*(RNAi), blue coloured line is *GFP/trr-2*(RNAi), red coloured line is *GFP/trr-1*(RNAi) and orange coloured line is *GFP/LPT*(RNAi). The *GFP/GFP*(RNAi) line overlaps with *GFP/trr-1*(RNAi) and *GFP/trr-2*(RNAi). n=10. Scale bars: 200 µm

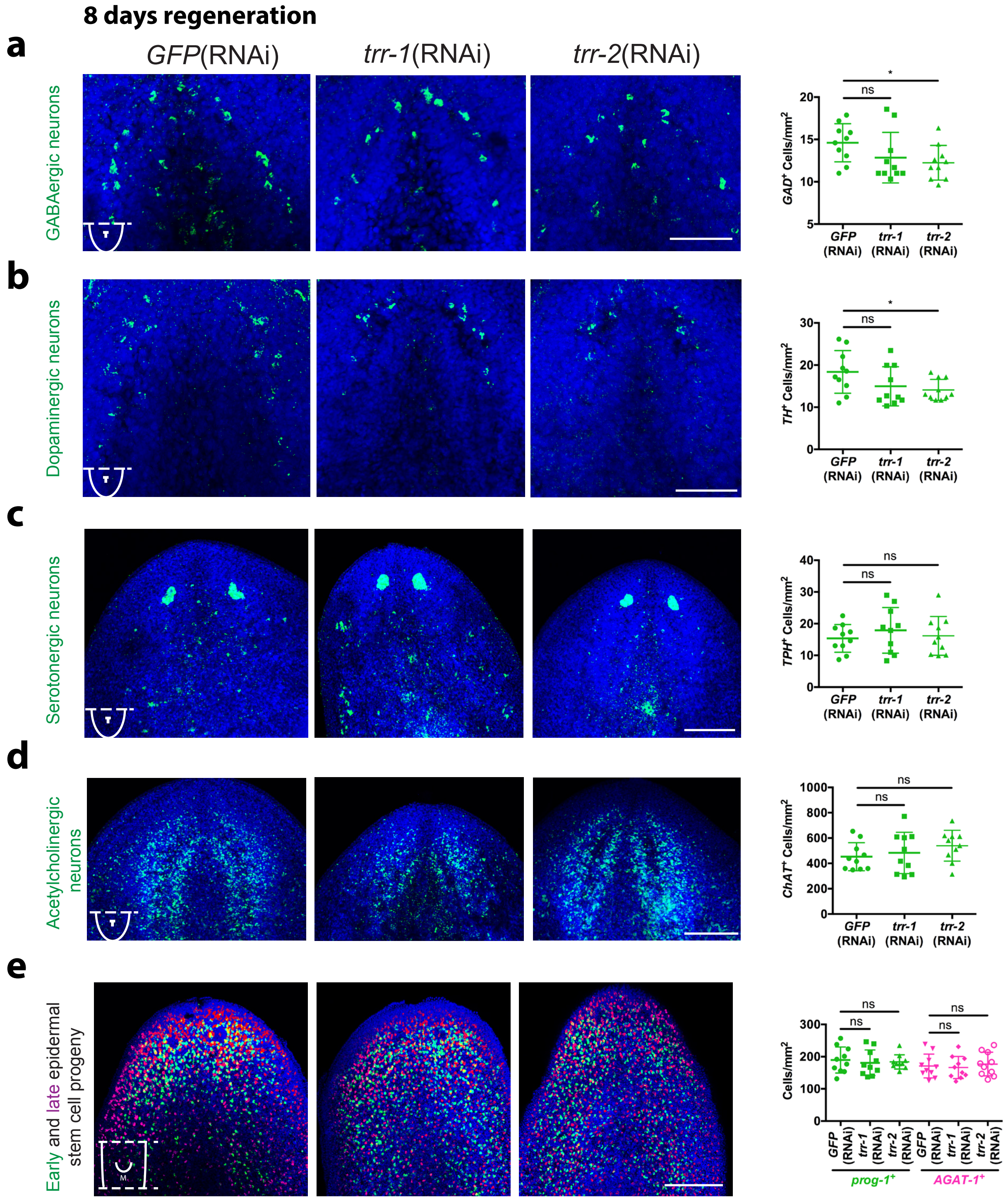

Supplementary Figure 3. ***Trr-2*****(RNAi) regenerating animals produce less GABAergic and dopaminergic neurons.**

a Quantification of the number of GABAergic neurons (labeled by *GAD*), **b** dopaminergic neurons (labeled by *TH*), **c** serotonergic neurons (labeled by *TPH*), **d** acetylcholinergic neurons (labeled by *chat*) and **e** early (labeled by *prog-1*) and late (labeled by *AGAT-1*) epidermal stem cell progeny at 8 days of regeneration of tail or middle pieces following *trr-1*(RNAi) or *trr-2*(RNAi). 2-tailed t-test used for analysis, n=10, *$p<0.05$, ns is not significant, lines and error bars indicate mean and SD. Scale bars: 200 µm

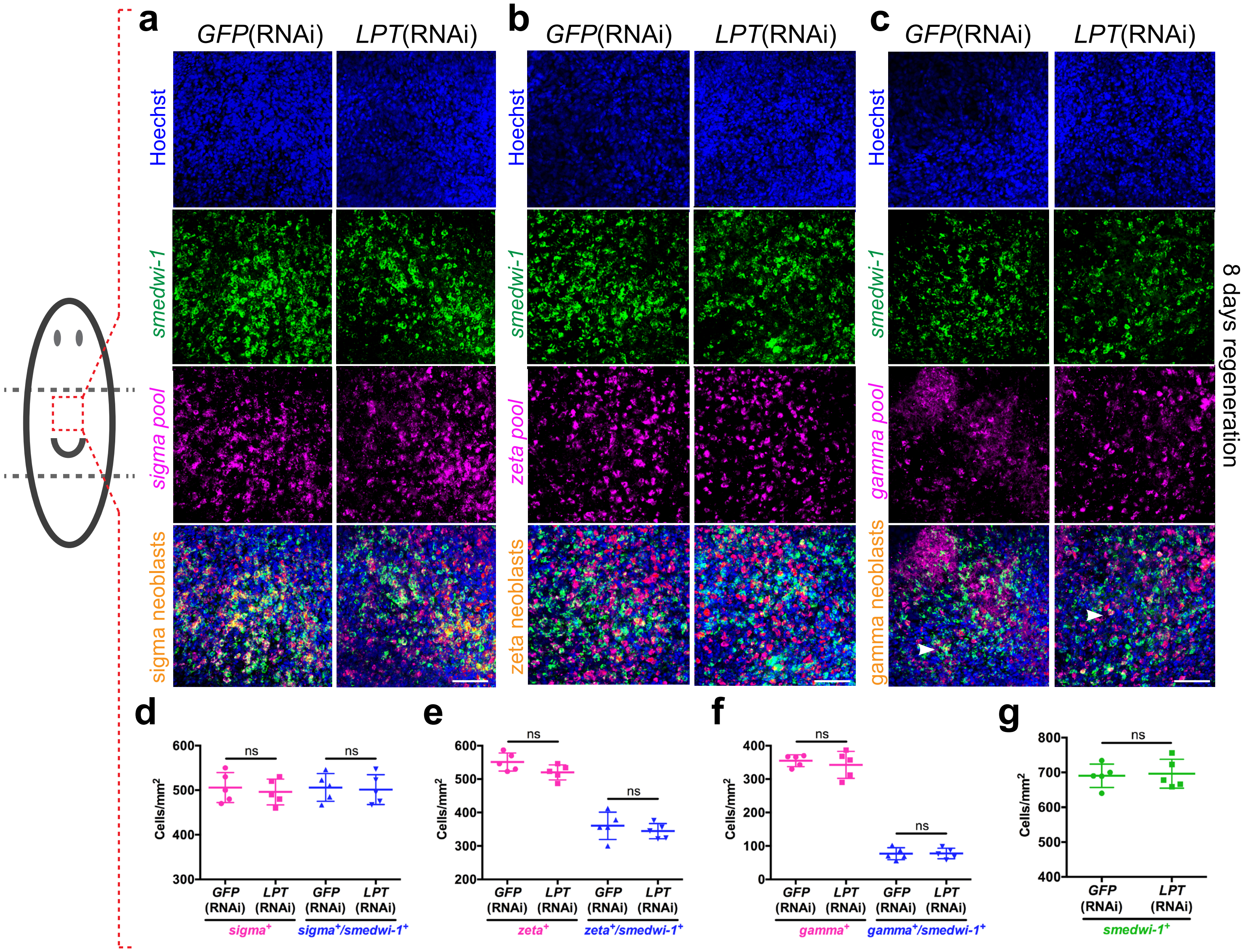

Supplementary Figure 4. *Sigma*, *zeta* and *gamma* neoblast numbers are unchanged following *LPT*(RNAi).

a FISH showing cells in 8 days of regenerating animals following *LPT*(RNAi) labeled by the *sigma pool* of RNA probes (*Soxp1*, *Soxp2*) and *smedwi-1*. White arrows point towards *sigma* neoblasts (double-positive for *sigma pool* and *smedwi-1*). **b** FISH showing cells in 8 days of regenerating animals following *LPT*(RNAi) labeled by the *zeta pool* of RNA probes (*zfp-1*, *Soxp3, egr-1*) and *smedwi-1*. White arrows point towards *zeta* neoblasts (double-positive for *zeta pool* and *smedwi-1*). **c** FISH showing cells in 8 days of regenerating animals following *LPT*(RNAi) labeled by the *gamma pool* of RNA probes (*gata4/5/6*, *hnf4*) and *smedwi-1*. White arrows point towards *gamma* neoblasts (double-positive for *gamma pool* and *smedwi-1*). **d-g** Graphs showing quantification of sigma (**d**), zeta (**e**), gamma (**f**) and total *smedwi-1*$^+$ (**g**) neoblasts in 8-day regenerating animals following *LPT*(RNAi). Each dot represents average number of cells in a single worm (n=5). Lines and error bars indicate mean and SD. Student's t test: *$p<0.05$, ns is not significant. Scale bars: 50 µm

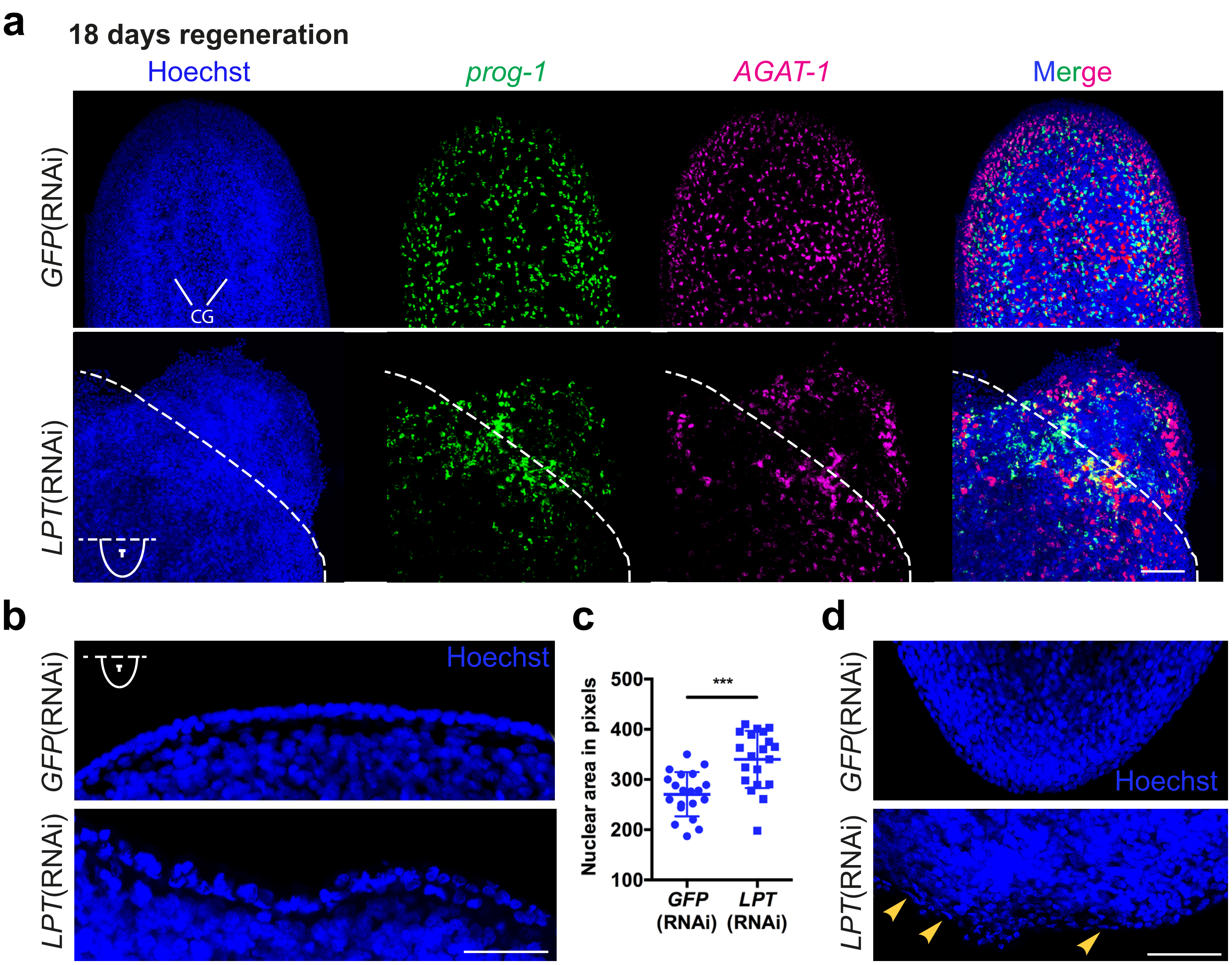

Supplementary Figure 5. *LPT*(RNAi) results in disorganized outgrowth-focused expression of epidermal precursor markers, epithelial disarray, hypertrophy and changes of nuclear morphology.

a Anterior part (containing an outgrowth) of a tail piece at 18 days of regeneration following *LPT*(RNAi) labeled with *prog-1* and *AGAT-1* epidermal precursor markers. 'CG' stands for 'cephalic ganglia'. **b** The epidermal layer (stained with Hoechst 33342) of a tail piece at 10 days of regeneration following *LPT*(RNAi) compared to control. **c** Graph showing increase in nuclear area following *LPT*(RNAi). Two-tailed t-test used for analysis, lines and error bars indicate mean and SD, n=20, ***p<0.001. **d** Image showing changes in nuclear morphology of epidermal cells in 10-day regenerating animals following *LPT*(RNAi). Nuclei were stained with Hoechst 33342. Yellow arrows point towards misshapen nuclei. Scale bars: 100 µm

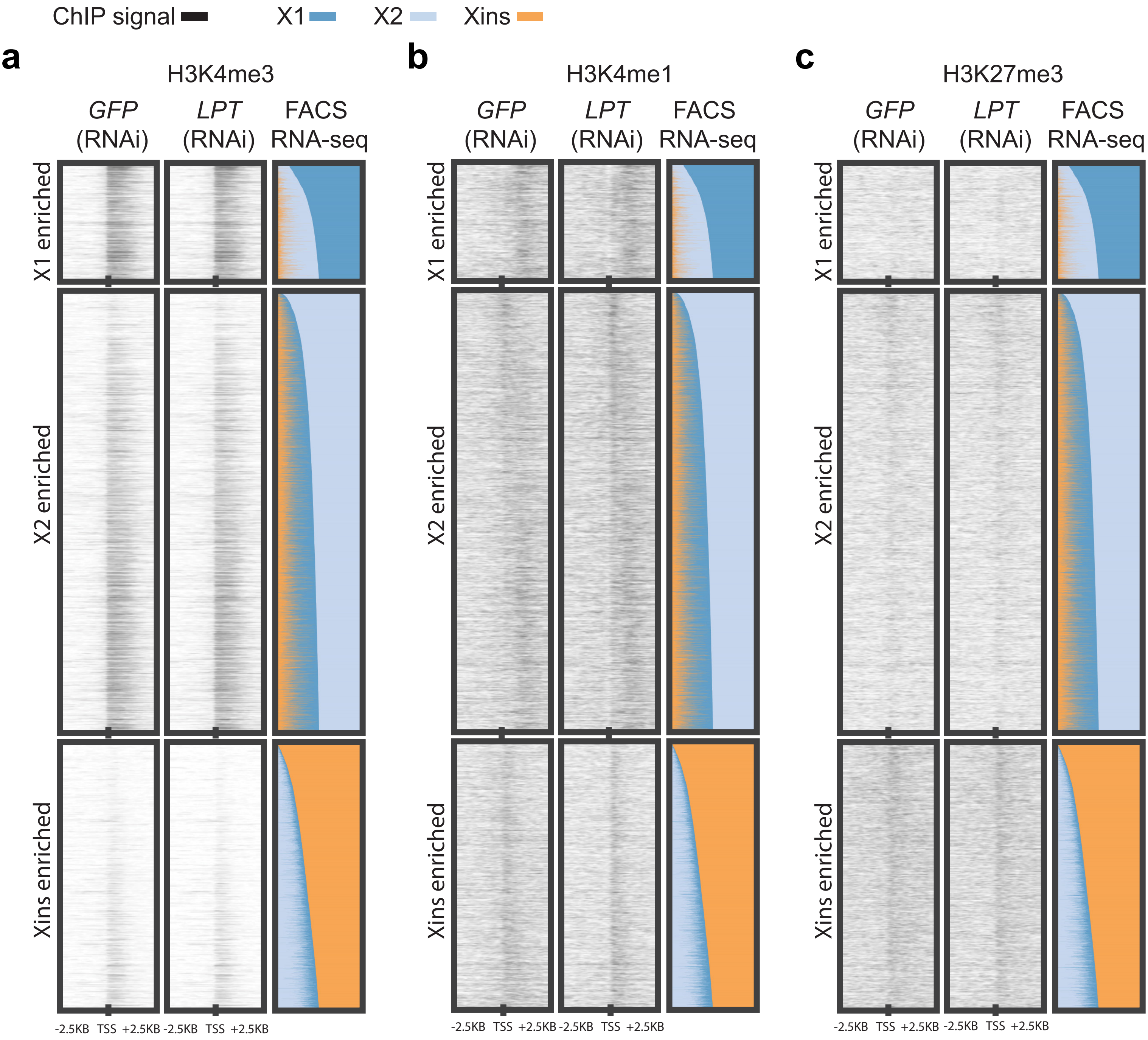

Supplementary Figure 6. Histone modification ChIP-seq profiles at promoter-proximal regions of different classes of genes.

a-c Images show histone modification patterns for H3K4me3 (**a**), H3K4me1 (**b**) and H3K27me3 (**c**) respectively. ChIP-seq signal is shown in black. Three classes of genes are presented – enriched >50% in X1 (G2/M stem cells) shown by dark blue, enriched >50% in X2 (G1 stem cells and stem cell progeny) shown in light blue, enriched >50% in X ins (differentiated cells) shown in orange. Histone modification graphs are centered on the Transcriptional Start Site (TSS) with 2.5 kb shown upstream and downstream.

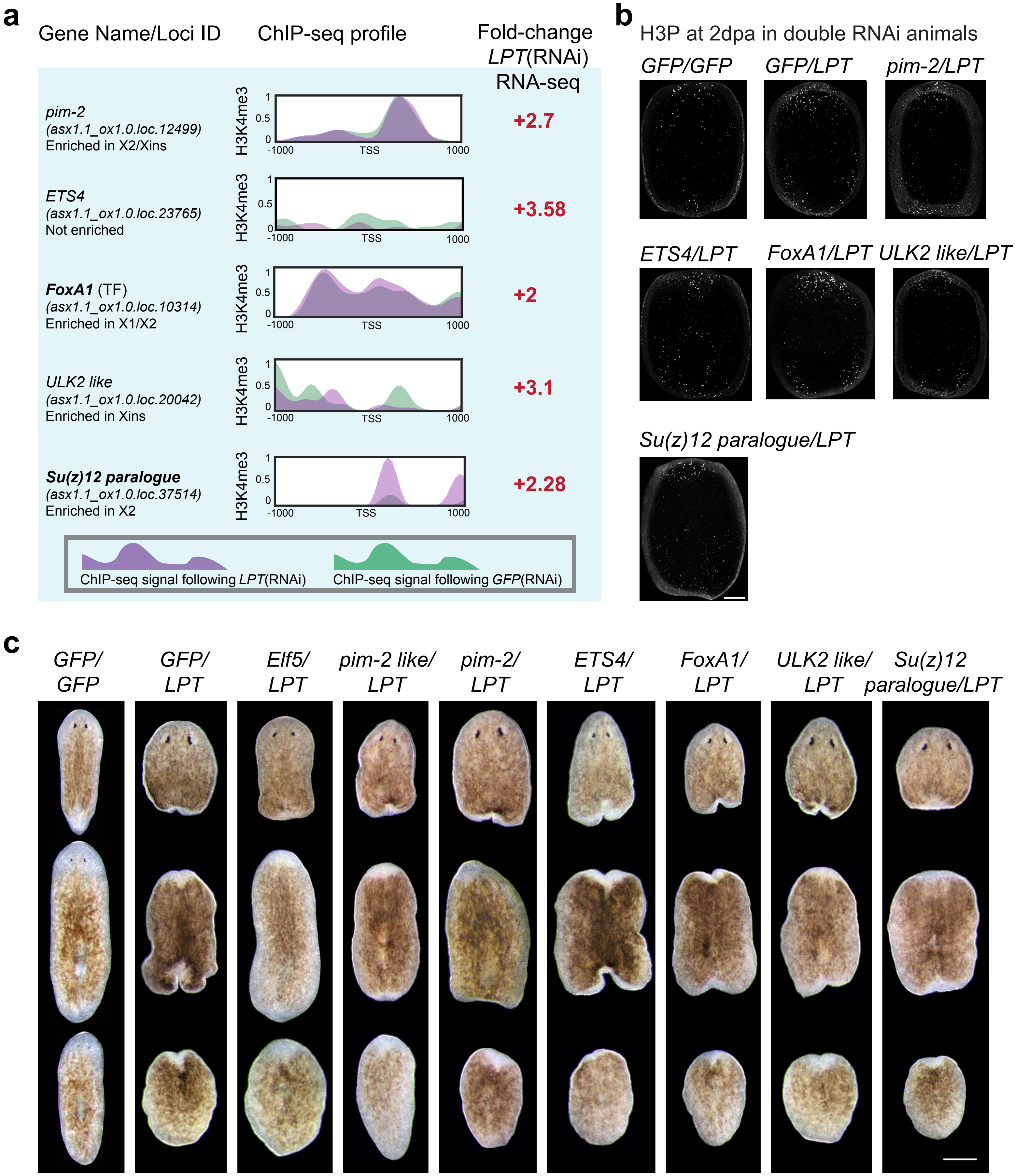

Supplementary Figure 7. Simultaneous knockdown of LPT with Elf5 or pim-2 like results in partial recovery of the LPT(RNAi) regenerative phenotype.

a Examples of genes significantly ($p<0.05$) up-regulated in G2/M stem cells following *LPT*(RNAi). The ChIP-seq profile for H3K4me3 in the 2 kb region around the TSS of each gene is presented. Purple

colour represents normalised signal following *LPT*(RNAi) and green colour is used to show the normalised signal following *GFP*(RNAi). 'TF' stands for 'transcription factor'. Bold font of a gene name illustrates an example where there is a correlation between H3K4me3 profile and up-regulation in RNA-seq data. **b** Representative examples of mitotic cells (labeled by anti-H3P antibody) in double RNAi condition at 48h post amputation. Scale bar: 50 μm. **c** Bright field images showing partial recovery in 10 day regenerating animals following *Elf5/LPT*(RNAi) and *pim-2 like/LPT*(RNAi) compared to *GFP/LPT*(RNAi). Other genes screened failed to recover the regeneration defects. Scale bar: 200 μm

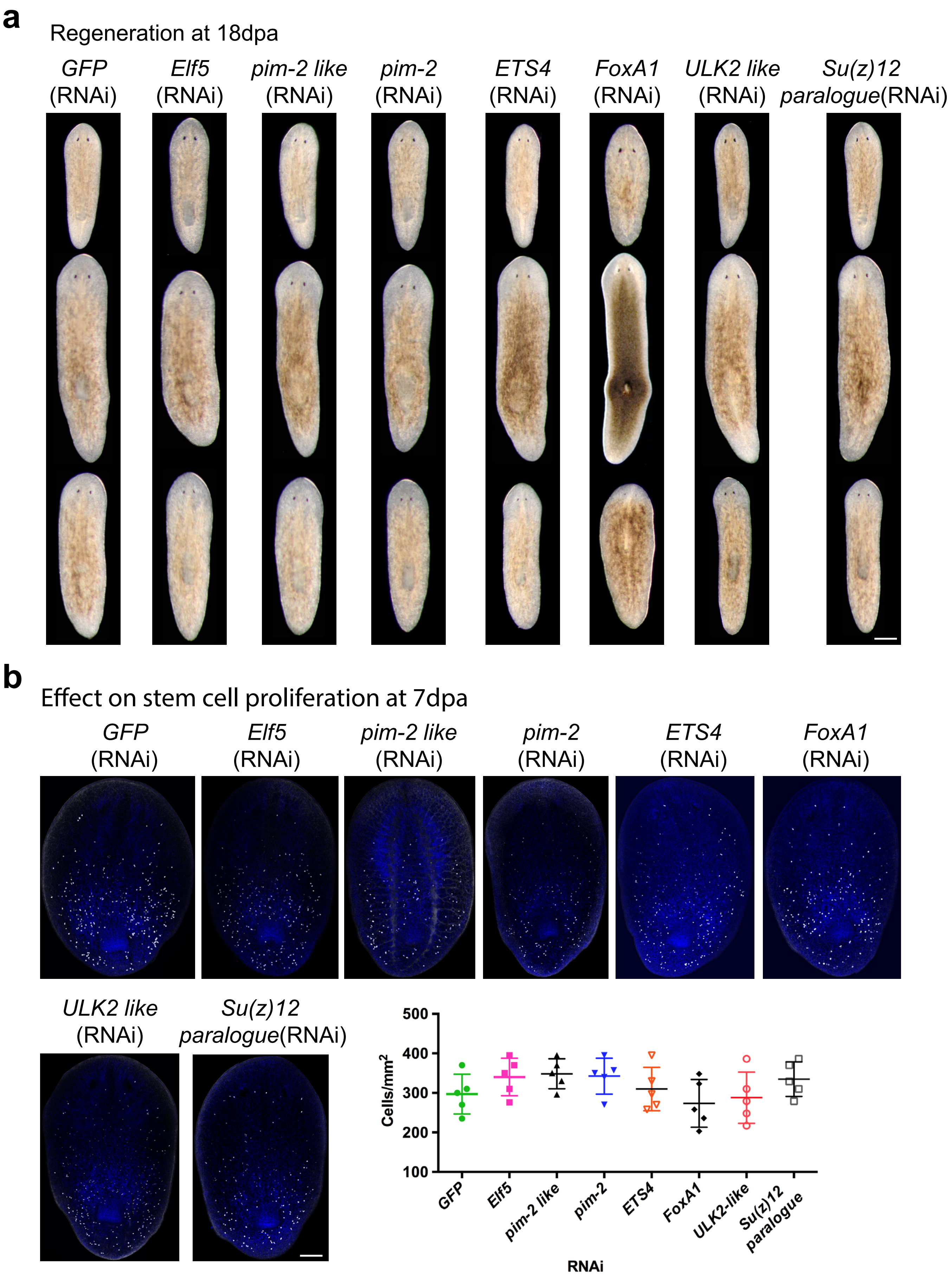

Supplementary Figure 8. RNAi of genes up-regulated following *LPT*(RNAi) did not result in defects in regeneration or stem cell proliferation.

a Bright field images showing regeneration following RNAi of different genes up-regulated in *LPT*(RNAi). Scale bar: 300 µm. **b** Images showing mitotic cells (labeled by anti-H3P antibody) in regenerating animals following knockdown of different genes up-regulated in *LPT*(RNAi). Graph showing mitotic cell quantification following gene knockdowns. Scale bar: 100µm

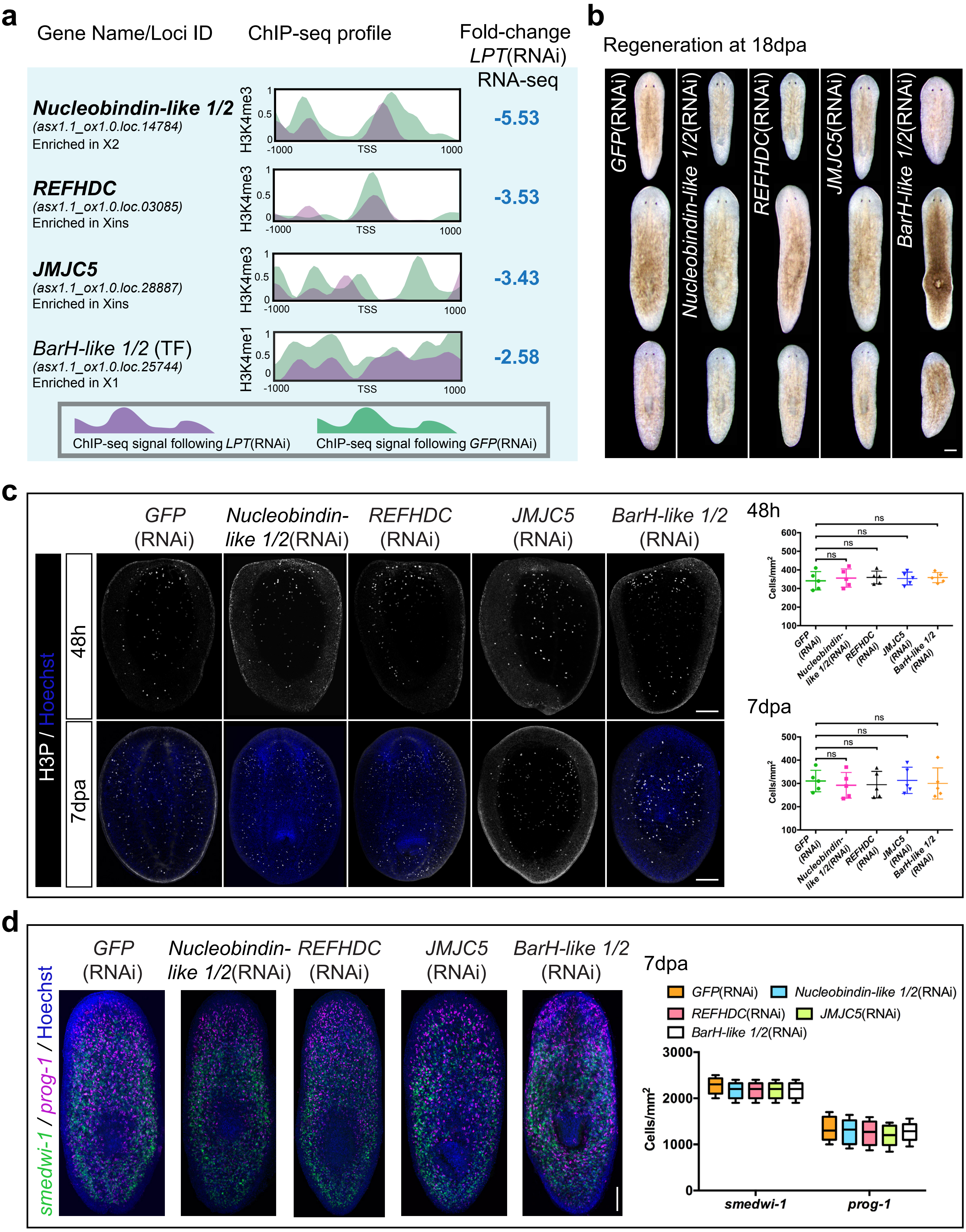

a
Gene Name/Loci ID
ChIP-seq profile
Fold-change LPT(RNAi) RNA-seq
Nucleobindin-like 1/2
(asx1.1_ox1.0.loc.14784)
Enriched in X2
-5.53
REFHDC
(asx1.1_ox1.0.loc.03085)
Enriched in Xins
-3.53
JMJC5
(asx1.1_ox1.0.loc.28887)
Enriched in Xins
-3.43
BarH-like 1/2 (TF)
(asx1.1_ox1.0.loc.25744)
Enriched in X1
-2.58
H3K4me3
H3K4me1
-1000
TSS
1000
ChIP-seq signal following LPT(RNAi)
ChIP-seq signal following GFP(RNAi)
b
Regeneration at 18dpa
GFP(RNAi)
Nucleobindin-like 1/2(RNAi)
REFHDC(RNAi)
JMJC5(RNAi)
BarH-like 1/2(RNAi)
c
GFP (RNAi)
Nucleobindin-like 1/2(RNAi)
REFHDC (RNAi)
JMJC5 (RNAi)
BarH-like 1/2 (RNAi)
H3P / Hoechst
48h
7dpa
ns
Cells/mm²
d
GFP (RNAi)
Nucleobindin-like 1/2(RNAi)
REFHDC (RNAi)
JMJC5 (RNAi)
BarH-like 1/2 (RNAi)
smedwi-1 / prog-1 / Hoechst
7dpa
GFP(RNAi)
Nucleobindin-like 1/2(RNAi)
REFHDC(RNAi)
JMJC5(RNAi)
BarH-like 1/2(RNAi)
Cells/mm²
smedwi-1
prog-1

Supplementary Figure 9. Knockdown of some genes down-regulated following LPT(RNAi) does not result in regeneration defects.

a Examples of genes significantly ($p<0.05$) down-regulated in G2/M stem cells following *LPT*(RNAi). The ChIP-seq profile for H3K4me3 and H3K4me1 in the 2 kb region around the TSS of each gene is presented. Purple colour represents normalised signal following *LPT*(RNAi) and green colour is used to show the normalised signal following *GFP*(RNAi). 'TF' stands for 'transcription factor'. Bold font of a gene name illustrates an example where there is a correlation between H3K4me3 profile and down-regulation in RNA-seq data. **b** Bright field images of 18-day regenerating animals following RNAi of different genes down-regulated in *LPT*(RNAi). **c** Representative examples of mitotic cells (labeled by anti-H3P antibody) at 48h and 7 day post amputation in regenerating animals following knockdown of different genes down-regulated in LPT(RNAi). Graphs show the quantification of mitotic cells. Each dot represents average number of mitotic cells in a single worm (n=5). Lines and error bars indicate mean and SD. Student's t test was used for analysis. **d** Representative FISH images showing stem cells (*smedwi-1*$^{+}$) and early epidermal progeny (*prog-1*$^{+}$) in 7 day regenerating animals in different RNAi conditions. Graph shows the quantification of *smedwi-1*$^{+}$ and *prog-1*$^{+}$ cells (n=5). Lines and error bars indicate mean and SD. Student's t test used for analysis, ns is not significant. Scale bars: 100 μm.

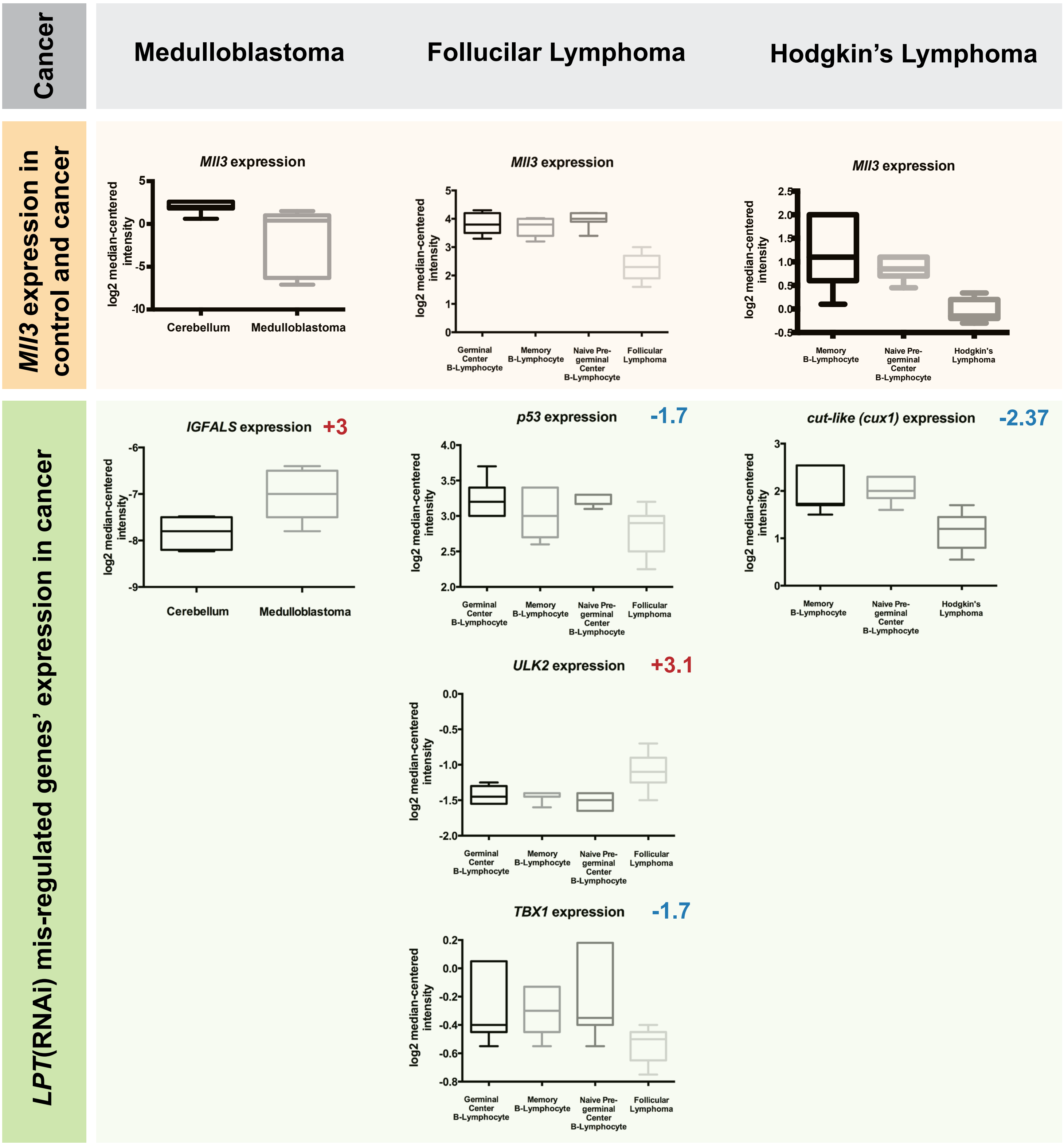

Red and blue numbers indicate fold changes of up- (red) and down-regulated (blue) genes in *LPT*(RNAi) X1 cells

Supplementary Figure 10. Mis-regulation of genes following *LPT*(RNAi) correlates with mis-regulation in human cancers where *Mll3* levels are decreased.

In silico analysis (www.oncomine.org; t-test, $p<0.0001$) of *Mll3*, *IGFALS, p53, ULK2, TBX1* and *cut-like* expression in normal tissue (cerebellum or different B-lymphocyte types) and cancer tissue (medulloblastoma, follicular lymphoma or Hodgkin's lymphoma). Positive and negative numbers next to gene names indicate fold up- or down-regulation in *LPT*(RNAi) X1 RNA-seq respectively. Lines and error bars indicate mean and SD.

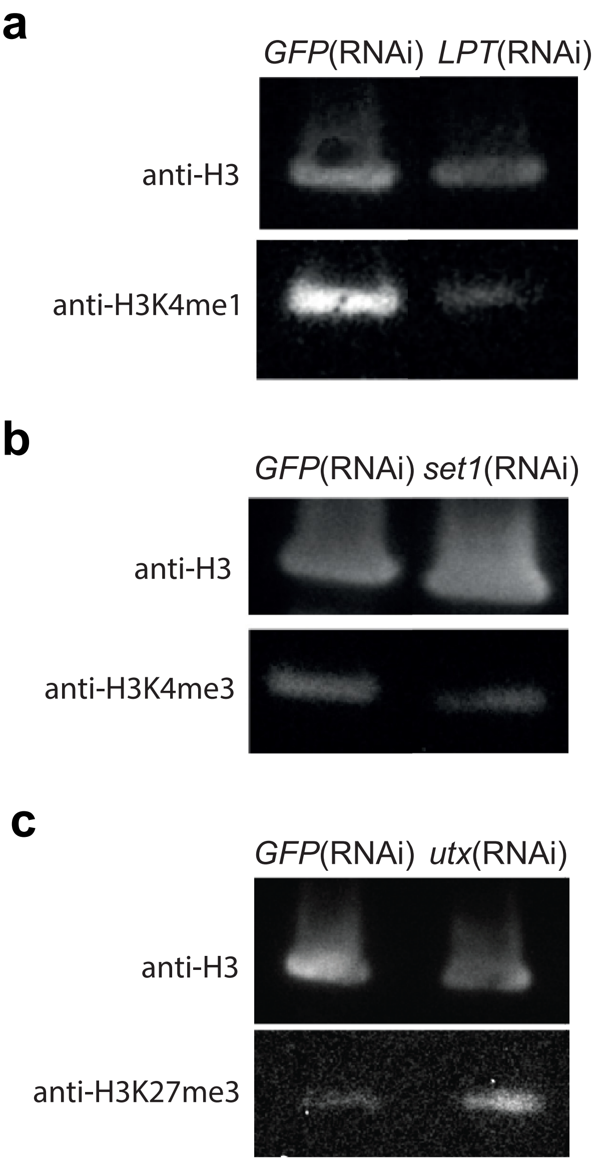

Supplementary Figure 11. The histone modifications antibodies used for ChIP-seq experiments are specific.

a Western blot with anti-H3K4me1 and loading control anti-H3 (unmodified histone H3) on protein lysate from *GFP*(RNAi) and *LPT*(RNAi) animals. **b** Western blot with anti-H3K4me3 and loading control anti-H3 (unmodified histone H3) on protein lysate from *GFP*(RNAi) and *set1*(RNAi) animals. **c** Western blot with anti-H3K27me3 and loading control anti-H3 (unmodified histone H3) on protein lysate from *GFP*(RNAi) and *utx(RNAi)* animals.

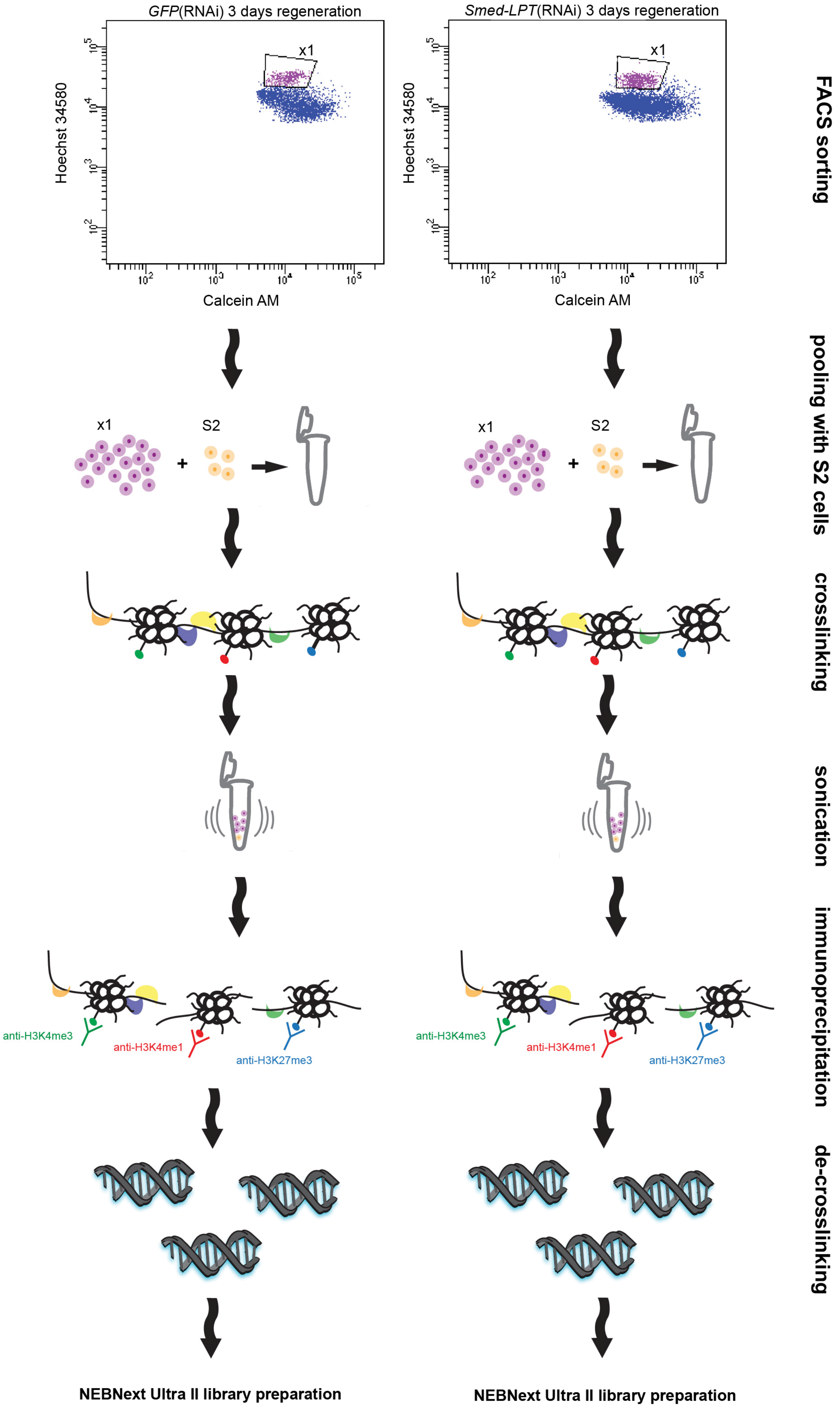

GFP(RNAi) 3 days regeneration
Smed-LPT(RNAi) 3 days regeneration
x1
Hoechst 34580
Calcein AM
FACS sorting
x1
S2
pooling with S2 cells
crosslinking
sonication
anti-H3K4me3
anti-H3K4me1
anti-H3K27me3
immunoprecipitation
de-crosslinking
NEBNext Ultra II library preparation

Supplementary Figure 12. Summary of planarian ChIP-seq procedure. Three day-regenerating planarians were dissociated into single cells. Cells were stained with Hoechst 34580 and Calcein AM in order to visualize cell populations according to nuclear size and cytoplasmic complexity. The X1 (G2/M) stem cells (magenta) were sorted and mixed with 4% *Drosophila* S2 cells. Cells were crosslinked with 1% Formaldehyde and sonicated. Immunoprecipitation with anti-H3K4me3, anti-H3K4me1 and anti-H3K27me3 antibodies followed. Samples were reverse-crosslinked and libraries were prepared using NEBNext Ultra II library preparation kit.

Primer name	Sequence (5'-3')	Author/publication	Use
Smed-H2B_F	TCTGTTAA GAAGATTTCAAAGG	Solana et al., 2012	RNA probe, dsRNA
Smed-H2B_R	TCCTGTG TATTTTGTAACAGC	Solana et al., 2012	RNA probe, dsRNA
Smed-porcupine-1_F	CCAGATG CAATCAAGTTTTTCGG	Jaber-Hijazi et al., 2013	RNA probe
Smed-porcupine-1_R	AACCGATA TTGGGCTTTTCTGG	Gurley et al., 2008	RNA probe
Smed-LPT_F	CAATGGT GACGACCCAGAAATC	Farah Jaber-Hijazi	RNA probe (pooled)
Smed-LPT_R	GGCTCAAT ATCAGTGGCGGTTC	Farah Jaber-Hijazi	RNA probe (pooled)
Smed-LPT_F2	CAACAATTACACTCTCT GTGACC	Yuliana Mihaylova	RNA probe (pooled)
Smed-LPT_R2	CAAGATCATCATCTGC AATCTCC	Yuliana Mihaylova	RNA probe (pooled)
Smed-trr-1_F	CGGATCGACTTGTTCA GAAACC	Yuliana Mihaylova	RNA probe (pooled)
Smed-trr-1_R	CCGATATTGTTGCCAT TTAC	Yuliana Mihaylova	RNA probe (pooled)
Smed-trr-1_F2	GTTGTCTGTGTTTCATT GTCC	Yuliana Mihaylova	RNA probe (pooled)
Smed-trr-1_R2	AGTGCTGTTGATTTGT AGACC	Yuliana Mihaylova	RNA probe (pooled)
Smed-trr-2_F	GGACGTATGTGAGAC GAATG	Yuliana Mihaylova	RNA probe (pooled)
Smed-trr-2_R	GATCCGACTTCCACAA TCCAGTGA	Yuliana Mihaylova	RNA probe (pooled)
Smed-trr2_F2	AACAATTACCGACATC TTCC	Yuliana Mihaylova	RNA probe (pooled)
Smed-trr2_R2	TTGATCAGGGTAAACA TTCC	Yuliana Mihaylova	RNA probe (pooled)
Smed-trr-2_F3	TGTGTGTTATGTAGGA ATGTGG	Yuliana Mihaylova	RNA probe (pooled)
Smed-trr2_R3	GTTGAATGATAACCGA CTGG	Yuliana Mihaylova	RNA probe (pooled)
Smed-GAD_F	ACGATTGCCGTA GCTGGAGA	Yuliana Mihaylova	RNA probe
Smed-GAD_R	ATGAAGCCATAT GCCAAAGC	Yuliana Mihaylova	RNA probe
Smed-TH_F	TGCACTAGAAG GTCTTACAAGG	Yuliana Mihaylova	RNA probe
Smed-TH_R	GGACCATATTCA CACTCCTCTT	Yuliana Mihaylova	RNA probe
Smed-TPH_F	CGTTCAAGAGGT CGACATGA	Yuliana Mihaylova	RNA probe
Smed-TPH_R	TACACGGAATGC AAGACCAG	Yuliana Mihaylova	RNA probe
Smed-chat_F	CATCCCTTGCAC TAAAATTTCC	Yuliana Mihaylova	RNA probe
Smed-chat_R	ATAAGGTTGTTG GTGTGATTGC	Yuliana Mihaylova	RNA probe
Smed-pitx_F	GTCATTCTCCAT CGGCTCAT	Marz et al., 2013	RNA probe
Smed-pitx_R	TGACAACATTGG CTGTCGAT	Marz et al., 2013	RNA probe
Smed-laminin_F	TGATGCGTGGT ATC	Jaber-Hijazi et al., 2013	RNA probe
Smed-laminin_R	TCGCTGGCAAAG TG	Jaber-Hijazi et al., 2013	RNA probe
Smed-CAVII-1_F	CTCTCGAAAGAC ATGAAACC	Yuliana Mihaylova	RNA probe
Smed-CAVII-1_R	TTGGTCTGAAAT TGTTCTCC	Yuliana Mihaylova	RNA probe
Smed-SP6-9_F	TTCAATAAATAA CGTTGAGAGCAA	Yuliana Mihaylova	RNA probe
Smed-SP6_9_R	TTCACAATTGTTT GTTAACGACTC	Yuliana Mihaylova	RNA probe
Smed-AGAT-1_F	GAAATGATTGA GTC CACCATGA	Eisenhoffer et al., 2008	RNA probe
Smed-AGAT-1_R	TGCAATATCTGG AT AAGGAGCA	Eisenhoffer et al., 2009	RNA probe
Smed-NB.21.11e_F	GTGATTGCGTTC GC GTATATT	Solana et al., 2012	RNA probe
Smed-NB.21.11e_R	ATTTATCCAGCG CG TCATATTC	Solana et al., 2012	RNA probe

Smedwi-1_F	TATTGACTCAGTGTGTGACG	Prasad Abnave	RNA probe
Smedwi-1_R	TCTGTCATTTATCGCTGGAG	Prasad Abnave	RNA probe
Smed-SoxP1 _F1	GCTTAGTGAAAAGGCCAAAG	Prasad Abnave	RNA probe (pooled)
Smed-SoxP1_R1	GCTTGATCTTCTACCGTCTT	Prasad Abnave	RNA probe (pooled)
Smed-SoxP1 _F2	GGCTCAATTACATGCTCTTG	Prasad Abnave	RNA probe (pooled)
Smed-SoxP1_R2	GTCCTGTGATCCCATAATCC	Prasad Abnave	RNA probe (pooled)
Smed-SoxP2_F1	CCCCATTAATTCCACACCTT	Prasad Abnave	RNA probe (pooled)
Smed-SoxP2_R1	GGAATCGCTAGTGTGTTTTG	Prasad Abnave	RNA probe (pooled)
Smed-SoxP2_F2	CAAAACACACTAGCGATTCC	Prasad Abnave	RNA probe (pooled)
Smed-SoxP2_R2	TAAACGACCAAGGAGAACTG	Prasad Abnave	RNA probe (pooled)
Smed-zfp1_F1	GCCAGTTTTATTTCACCAGG	Prasad Abnave	RNA probe (pooled)
Smed-zfp1_R1	GATCTTTGAGTGAAGCTGGT	Prasad Abnave	RNA probe (pooled)
Smed-zfp1_F2	CTCTTCAAGTACATGGCAGT	Prasad Abnave	RNA probe (pooled)
Smed-zfp1_R2	TATCTACGTCCATGGAGTCA	Prasad Abnave	RNA probe (pooled)
Smed-SoxP3_F	GAAATGTCAGCTGAAGAACG	Prasad Abnave	RNA probe (pooled)
Smed-SoxP3_R	CGGATGACTGTTGTAGATCA	Prasad Abnave	RNA probe (pooled)
Smed-gata4/5/6_F1	GATTGCACAACAGACATCAG	Prasad Abnave	RNA probe (pooled)
Smed-gata4/5/6_R1	TATTGCTAGCTCCACAGTTC	Prasad Abnave	RNA probe (pooled)
Smed-gata4/5/6_F2	GAACTGTGGAGCTAGCAATA	Prasad Abnave	RNA probe (pooled)
Smed-gata4/5/6_R2	TGAAGGGCTGGACTGATATA	Prasad Abnave	RNA probe (pooled)
Smed-gata4/5/6_F3	GGAGAAGAAACAACGAAGGA	Prasad Abnave	RNA probe (pooled)
Smed-gata4/5/6_R3	GGGGCTCATCTTTGATTTTG	Prasad Abnave	RNA probe (pooled)
Smed-hnf4_F3	CTTCTCTGTTTTCATGCGTC	Prasad Abnave	RNA probe (pooled)
Smed-hnf4_R3	AATACCCGGCCTATTTCATC	Prasad Abnave	RNA probe (pooled)
Smed-utx_F	GGCTGCTTGGACAAATCTTGGTGT	Hubert et al., 2013	dsRNA
Smed-utx_R	GCACAGTTGAGGCAACGAGTTTCA	Hubert et al., 2013	dsRNA
Smed-Elf5_F	ACTCCAAGACTCGATGAGAAC	Yuliana Mihaylova	dsRNA
Smed-Elf5_R	TGAGTTGCGAAACCACTATC	Yuliana Mihaylova	dsRNA
Smed-pim-2-like_F	GAATATATTGGCGGTGATTG	Yuliana Mihaylova	dsRNA
Smed-pim-2-like_R	ACTCTGTGATGAATGATCTGG	Yuliana Mihaylova	dsRNA
Smed-PRDM1-1_F	CATTAAGTTACAATCGCTCTCC	Yuliana Mihaylova	dsRNA
Smed-PRDM1-1_R	TAATCGAATCTGTTGCTTCC	Yuliana Mihaylova	dsRNA
Smed-cut-like1_F	TAACTGATGAGGTGACTGATTG	Yuliana Mihaylova	dsRNA
Smed-cut-like1_R	ATTGGTAGAGTTGTCTGGTTTG	Yuliana Mihaylova	dsRNA
Smed-RREBP1_F	CAATTCATATCTTCGTCACCTC	Yuliana Mihaylova	dsRNA
Smed-RREBP1_R	ACTCGAGTCATATGTCGTTCTC	Yuliana Mihaylova	dsRNA
Smed-pim-2_F	ACATCCTGTTTGCACATTTC	Yuliana Mihaylova	dsRNA
Smed-pim-2_R	TCTTTCCATCACAAAACACC	Yuliana Mihaylova	dsRNA
Smed-ETS4_F	CGTCAATAAGTCAATGAAGAGG	Yuliana Mihaylova	dsRNA
Smed-ETS4_R	CAGAAATTGAGAATGGGGTAG	Yuliana Mihaylova	dsRNA

Smed-FoxA1_F	TGTTAGTCAGAGTTGTTCGATG	Yuliana Mihaylova	dsRNA
Smed-FoxA1_R	AGAACGGATTACTTGTCGTTAG	Yuliana Mihaylova	dsRNA
Smed-ULK2like_F	GTAGCCGTGAAAGTACCAAAG	Yuliana Mihaylova	dsRNA
Smed-ULK2like_R	CGAGGTCATGTACGACAAAG	Yuliana Mihaylova	dsRNA
Smed-Su(z)12paralogue_F	CAGATGTGCAAAATCAATCC	Yuliana Mihaylova	dsRNA
Smed-Su(z)12paralogue_R	ACTCAACAGACCAAATTCCAC	Yuliana Mihaylova	dsRNA
Smed-Nucleobindin-like1/2_F	TCCTACACATTAACTGCAACAC	Yuliana Mihaylova	dsRNA
Smed-Nucleobindin-like1/2_R	TCTAGCTCCTTCCTCAATCTC	Yuliana Mihaylova	dsRNA
Smed-REFHDC_F	CAATTGGGACACATATCACTAC	Yuliana Mihaylova	dsRNA
Smed-REFHDC_R	CTTCTAGCATGTCCTTTTCTTC	Yuliana Mihaylova	dsRNA
Smed-JMJC5_F	AGGTGCCATTGATACAAAAC	Yuliana Mihaylova	dsRNA
Smed-JMJC5_R	CTGTGTCCAATTCAGAATAACC	Yuliana Mihaylova	dsRNA
Smed-BarH-like1/2_F	GTTGATCCAGTCAACAGTCC	Yuliana Mihaylova	dsRNA
Smed-BarH-like1/2_R	CAGCTTCAGCTAACAATTCC	Yuliana Mihaylova	dsRNA
M13 Forward	GTAAAACGACGGCCA G	Orientation of plasmid insert; RNA probe; dsRNA	
M13 Reverse	CAGGAAACAGCTATG AC	Orientation of plasmid insert; RNA probe; dsRNA	
**Gene sequences for which primer sequences are not available, but have been kindly provided in a plasmid by a third party (below)**			
**Gene name**	**Vector**	**Author/Publication**	**Use**
GFP	pGEM-T	Daniel Felix	dsRNA
Smedwi-2	pGEM-T	Mette Handberg- Thorsager	RNA probe

Supplementary Table 1. Primer sequences.

All primers are given in 5'->3' orientation. 'F' and 'R' stand for 'forward' and 'reverse' primer respectively.
