# Supplementary material for: Conservation of epigenetic regulation by the MLL3/4 tumour suppressor in planarian pluripotent stem cells
Source: Nat Commun. 2018 Sep 7;9:3633. doi: 10.1038/s41467-018-06092-6 (PMC6128892; doi:10.1038/s41467-018-06092-6)
Supplement: Supplementary file 2 — Description of Additional Supplementary Files [file 41467_2018_6092_MOESM2_ESM.docx]

**Description of Additional Supplementary Files**

File Name: Supplementary Data 1

Description: Differentially expressed loci following *LPT*(RNAi). Each row represents one locus that was differentially expressed with a p-value less than 0.05 and fold change <-1.5 or >1.5. The Wald’s test (as part of the Sleuth software) was used for assessing differential expression. The top BLAST hit (with evalue) and the common model organism top BLAST hit is also provided for each locus.

File Name: Supplementary Software

Description: Python Notebook. Provides details on the ChIP-seq and RNA-seq bioinformatics analyses.
