# Supplementary material for: Conservation of epigenetic regulation by the MLL3/4 tumour suppressor in planarian pluripotent stem cells
Source: Nat Commun. 2018 Sep 7;9:3633. doi: 10.1038/s41467-018-06092-6 (PMC6128892; doi:10.1038/s41467-018-06092-6)
Supplement: Supplementary file 4 — Supplementary Software [file 41467_2018_6092_MOESM4_ESM.html]

main


In [1]:

```
%%HTML
<!--This HTML code produces the below link for toggling code cells within this notebook-->
<script>
var code_show = true;
function code_toggle() {
    if (code_show){
        $('div.input').hide();
        $('#toggleCode').text('show raw python code');
    } else {
        $('div.input').show();
        $('#toggleCode').text('hide raw python code')
    }
    code_show = !code_show
} 
$( document ).ready(code_toggle);
</script>
<table width=100% style='border:2px solid white'>
    <tr>
        <td style='border:none'><b>Last Updated March-25-2017</b></td>
        <td style='border:none'>
            <p style='text-align:right'>
                <b><a id='toggleCode' onClick='code_toggle()'></a></b>
            </p>
        </td>
    </tr>
</table>
```

|  |  |
| --- | --- |
| **Last Updated March-25-2017** |  |

In [93]:

```
#import python libraries and useful native functions
import os,sys,gzip
import pandas as pd
import matplotlib.pyplot as plt
import numpy as np
import seaborn as sns
from ipy_table import *
import scipy.stats as ss

from collections import defaultdict, Counter
from itertools import combinations

%matplotlib inline
```

# Bioinformatic analysis¶

## Table of contents¶

1. RNA-seq analysis
   1. Library metrics
   2. Differential expression
   3. GO enrichment of up/down regulated smed-LPT-RNAi genes
   4. Enrichment of up/down regulated smed-LPT RNAi genes in FACS categories
2. ChIP-seq processing
   1. Read trimming
   2. Read Mapping
   3. Duplicate removal
   4. Separate S.mediterranea and D.melanogaster reads
   5. Predicted fragment size of single-end reads
   6. Convert to BED format
   7. Conver to "mid" format
3. ChIP-seq normalization
   1. Normalization to input
   2. Normalization across samples
   3. Generate comparable coverage tracks among all samples and inputs
4. ChIP-seq analysis
   1. Outlier identification and removal
   2. GFP vs LPT profiles for FACS categories
   3. ChIP-seq correlation to smed-LPT RNA-seq data

#### Appendix¶

1. Data sources used
2. Software used

In [108]:

```
%%HTML
<a id='sec01'></a>
```

## RNA-seq analysis¶

RNA-seq libraries of X1 cells under GFP (control) RNAi and X1 cells under smed-LPT RNAi were prepared in biological duplicates:

```
rna_aboobaker_x1_gfp_072h_01
rna_aboobaker_x1_gfp_072h_02
rna_aboobaker_x1_lpt_072h_01
rna_aboobaker_x1_lpt_072h_02
```

RNA-seq reads were trimmed with Trimmomatic:

```
java -jar trimmomatic-0.36.jar PE -threads 32 -phred33 libA libB libA.paired libA.unpaired libB.paired libB.unpaired LEADING:20 TRAILING:20 MINLEN:30 ILLUMINACLIP:/data3/bin/Trimmomatic-0.36/adapters/adapters.fa:2:30:12:1:false TOPHRED33 2> lib.log
```

The reference transcriptome used is from CITE, a re-annotation of the current asexual S.mediterranea genome.

TPM values were generated using Kallisto. Sleuth was used to calculate the normalization factors. TPM can be aggregated by gene by summing the TPM of individual transcripts of a gene.

#### Library metrics¶

All four libraries are 75bp pair-end reads.

```
library                          # reads
rna_aboobaker_x1_gfp_072h_01     49,044,260
rna_aboobaker_x1_gfp_072h_02     51,348,940
rna_aboobaker_x1_lpt_072h_01     68,721,175
rna_aboobaker_x1_lpt_072h_02     87,571,687
```

#### Differential expression¶

In [37]:

```
lptFile = open('data/lpt.rnaseq.results')
lptFile.next()
lpt_fc = []
lids = []
for line in lptFile:
    meta = line.strip().split()
    if meta[4] != 'NA':
        if float(meta[4]) <= 0.05:
            lids.append(meta[0])
            lpt_fc.append([float(meta[3])])

lpt_fc = pd.DataFrame(lpt_fc,columns=['fc'],index=lids)
lpt_fc['abs_fc'] = lpt_fc['fc'].abs()
lptFile.close()

lpt_down = set(lpt_fc[lpt_fc['fc'] < -0.58].index)
lpt_up = set(lpt_fc[lpt_fc['fc'] > 0.58].index)
```

In [91]:

```
propFile = open('data/facs_proportions.tab')
lids = []
props = {}
propIndex = []
df_props = []
for line in propFile:
    meta = line.strip().split()
    propIndex.append(meta[0])
    props[meta[0]] = map(float,meta[1:])
    df_props.append(map(float,meta[1:]))
propFile.close()
df_props = pd.DataFrame(df_props,index=propIndex,columns=['x1','x2','xins'])

all_lids = set(propIndex)
x1_lids = set([k for k,v in props.items() if v[0] >= 50.0])
x2_lids = set([k for k,v in props.items() if v[1] >= 50.0])
xins_lids = set([k for k,v in props.items() if v[2] >= 50.0])
x12_lids = set([k for k,v in props.items() if v[0] + v[1] >= 75.0]) - x1_lids - x2_lids - xins_lids
x2ins_lids = set([k for k,v in props.items() if v[1] + v[2] >= 75.0]) - x1_lids - x2_lids - xins_lids
x1ins_lids = set([k for k,v in props.items() if v[0] + v[2] >= 75.0]) - x1_lids - x2_lids - xins_lids
x0_lids = set(props.keys()) - x1_lids - x2_lids - xins_lids - x12_lids - x2ins_lids - x1ins_lids

tf_lids = set(open('data/tf.lids').read().strip().split('\n'))
```

In [40]:

```
print 'Number of genes down-regulated at fold-change >= 1.5:', len(lpt_down)
print 'Number of genes up-regulated at fold-change >= 1.5:', len(lpt_up)
```

```
Number of genes down-regulated at fold-change >= 1.5: 540
Number of genes up-regulated at fold-change >= 1.5: 542
```

#### FACS category enrichment¶

Enrichment of up/down-regulated smed-LPT RNAi loci in FACS categories.

In [95]:

```
def enrichment(popNum,condANum,condBNum,overlapNum):
    return ss.hypergeom.sf(overlapNum - 1,popNum,condANum,condBNum)
l = {'x1':x1_lids,'x2':x2_lids,'xins':xins_lids,\
     'x1/x2':x12_lids,'x2/xins':x2ins_lids,'unenriched':x0_lids,'tf':tf_lids}

enrichmentTable = []
row = [' ']
for name in ['x1','x2','xins','x1/x2','x2/xins','unenriched','tf']:
    row.append(name + ' (' + str(len(l[name])) + ')')
enrichmentTable.append(row)

row = ['<b>LPT up-regulated (' + str(len(lpt_up)) + ')</b>']
for name in ['x1','x2','xins','x1/x2','x2/xins','unenriched','tf']:
    d = l[name]
    e = enrichment(len(all_lids),len(lpt_up),len(d),len(lpt_up & d))
    ov = str(len(lpt_up & d))
    if e < 0.01:
        ov = '<b>' + ov + '</b>'
    row.append(ov + '\n' + str(e))
enrichmentTable.append(row)

row = ['<b>LPT down-regulated (' + str(len(lpt_down)) + ')</b>']
for name in ['x1','x2','xins','x1/x2','x2/xins','unenriched','tf']:
    d = l[name]
    e = enrichment(len(all_lids),len(lpt_down),len(d),len(lpt_down & d))
    ov = str(len(lpt_down & d))
    if e < 0.01:
        ov = '<b>' + ov + '</b>'
    row.append(ov + '<br>' + str(e))
enrichmentTable.append(row)
```

In [97]:

```
make_table(enrichmentTable)
apply_theme('basic')
```

Out[97]:

|  |  |  |  |  |  |  |  |
| --- | --- | --- | --- | --- | --- | --- | --- |
|  | **x1 (2253)** | **x2 (8444)** | **xins (5119)** | **x1/x2 (4538)** | **x2/xins (3652)** | **unenriched (2897)** | **tf (489)** |
| **LPT&nbspup-regulated (542)** | 49 0.279827085696 | 160 0.792766780395 | 86 0.968427783231 | 83 0.820796542146 | 79 0.230230754871 | 53 0.765849059846 | **18** 0.00986037739625 |
| **LPT&nbspdown-regulated (540)** | 37 0.905365926307 | 174 0.288389760354 | **155** 1.18487642609e-08 | 44 0.99999999827 | 76 0.345749652507 | 32 0.999960280215 | **29** 2.04472413024e-07 |

In [109]:

```
%%HTML
<a id='sec02'></a>
```

## ChIP-seq processing¶

The ChIP-seq libraries used in the analysis is listed in the appendix.

In addition, the following chip/rna-seq libraries were prepared. This neoblast analysis is only concerned with the GFP (control) libraries. The LPT RNAi libraries are to be used in a separate publication.

```
chip_aboobaker_x1_h3k27me3_gfp_na_01
chip_aboobaker_x1_h3k27me3_gfp_na_02
chip_aboobaker_x1_h3k27me3_gfp_na_03
chip_aboobaker_x1_h3k27me3_lpt_na_01
chip_aboobaker_x1_h3k27me3_lpt_na_02
chip_aboobaker_x1_h3k27me3_lpt_na_03
chip_aboobaker_x1_h3k27me3_na_na_01
chip_aboobaker_x1_h3k4me1_gfp_na_01
chip_aboobaker_x1_h3k4me1_gfp_na_02
chip_aboobaker_x1_h3k4me1_gfp_na_03
chip_aboobaker_x1_h3k4me1_lpt_na_01
chip_aboobaker_x1_h3k4me1_lpt_na_02
chip_aboobaker_x1_h3k4me1_lpt_na_03
chip_aboobaker_x1_h3k4me1_na_na_01
chip_aboobaker_x1_h3k4me3_gfp_na_01
chip_aboobaker_x1_h3k4me3_gfp_na_02
chip_aboobaker_x1_h3k4me3_gfp_na_03
chip_aboobaker_x1_h3k4me3_lpt_na_01
chip_aboobaker_x1_h3k4me3_lpt_na_02
chip_aboobaker_x1_h3k4me3_lpt_na_03
chip_aboobaker_x1_h3k4me3_na_na_01
chip_aboobaker_x1_input_gfp_na_01
chip_aboobaker_x1_input_gfp_na_02
chip_aboobaker_x1_input_gfp_na_03
chip_aboobaker_x1_input_lpt_na_01
chip_aboobaker_x1_input_lpt_na_02
chip_aboobaker_x1_input_lpt_na_03
chip_aboobaker_x1_input_na_na_01
```

The goal of the pre-processing step is to generate a .bed file representing genomic fragments that were sequenced in each of the chip-seq libraries.

Chip-seq libraris are available from the Aboobaker lab and Sanchez lab. Both labs used Drosophila spike in controls for down-stream normalization. The Aboobaker lab libraries are pair-end 40bp. The sanchez lab libraries are single-end 50bp.

The general steps of this process are:

1. Trim reads for quality
2. Map reads to the asexual S.mediterranea and D.melanogaster genome
3. Filter out ambiguous reads (low mapping quality, discordant pairs, PCR duplicates)
4. Separate out S.med / D.mel reads
5. Convert to .bed file

#### Read trimming¶

All reads trimmed with trimmomatic:

```
java -jar trimmomatic-0.36.jar PE -threads 32 -phred33 libA libB libA.paired libA.unpaired libB.paired libB.unpaired LEADING:20 TRAILING:20 MINLEN:30 ILLUMINACLIP:/data3/bin/Trimmomatic-0.36/adapters/adapters.fa:2:30:12:1:false TOPHRED33 2> lib.log
```

#### Read mapping¶

The S.mediterranea asexual genome (SmedAsxl\_genome\_v1.1.nt) was downloaded from smedgd database (http://smedgd.stowers.org/). The D. melanogaster genome (r6.10) was downloaded from flybase. The species name (Smed, Dmel) were prepended to each fasta header for easier species identification down-stream. Both fasta files were concatenated and indexed with BWA.

The trimmed reads were mapped to this combined genome index with BWA. The BWA results were piped to samtools for filtering out unmapped reads and mapping quality less than 10:

```
# for single-end reads
bwa mem -t 32 smed_dmel.fasta lib | samtools view -b -F 4 -q 10 - > lib.filtered.bam

# for pair-end reads, use a secondary filter script to filter by pairs.
# discard pairs where both reads have mapping quality less than 10.
# also discard pairs where reads map to separate reference contigs

import sys

pair = []
for line in sys.stdin:
        if line[0] == '@':
                print line.strip()
        else:
                meta = line.strip().split()
                pair.append(meta)

                if len(pair) == 2:
                        if int(pair[0][4]) >= 10 or int(pair[1][4]) >= 10:
                                if pair[0][6] == '=' and pair[1][6] == '=':        
                                        print '\t'.join(pair[0])
                                        print '\t'.join(pair[1])

                        pair = []
```

```
bwa mem -t 32 smed_dmel.fasta libA.paired libB.paired | samtools view -h -f 2 - | python pe_filterMQ.py | samtools view -b - > lib.filtered.bam
```

The mapping results were sorted with samtools:

```
samtools sort -m 500M -T lib.tmp -O bam -@ 32 lib.filtered.bam > lib.filtered.sorted.bam
```

#### Duplicate removal¶

Picard tools' MarkDuplicates was used to remove PCR duplicates:

```
java -jar picard.jar MarkDuplicates MAX_FILE_HANDLES=100 INPUT=lib.filtered.sorted.bam OUTPUT=lib.filtered.sorted.markdup.bam METRICS_FILE=lib.filtered.sorted.log REMOVE_DUPLICATES=true
```

#### Separate S.mediterranea and D.melanogaster into individual BAM files¶

Reads that were mapped to S.mediterranea were extracted from each bam file. Only reads where the forward and reverse both map to the same reference contig were kept. The prepended species name are removed during this step:

```
import sys

for line in sys.stdin:
    if line[0] == '@':
        if line[:3] == '@SQ':
            meta = line.strip().split()
            ref = meta[1].split(':')[1]
            if ref.split('_')[0] != 'dmel':
                meta[1] = 'SN:' + ref.split('_')[1]
                print '\t'.join(meta)
    else:
        meta = line.strip().split('\t')
        if meta[2].split('_')[0] != 'dmel':
            if meta[6] == '=' or meta[6] == '*':
                meta[2] = meta[2].split('_')[1]
                print '\t'.join(meta)
```

```
samtools view -h lib.filtered.sorted.markdup.bam | python filterDmel.py | samtools view -b > lib.filtered.sorted.markdup.smed.bam
```

#### Predict fragment size on single-end reads¶

Sanchez's chip-seq libraries were single-end. Use MACS2's predictd function to predict fragment sizes:

```
macs2 predictd -i file.bam 2> log
```

#### Convert to BED format¶

Each BAM file was converted to BED format. The BED file reflects the DNA fragment that was sequenced, not the sequenced read. So for single-end reads, an extension by mean fragment size needs to be performed to get the end coordinate. For pair-end reads, the position from the start of the forward read to the end of the reverse read is recorded in the BED file.

For single-end files, each read was extended from its respective orientation from 5` - 3` direction using the estimated fragment size generated previously. The resulting genomic interval is then reported in the BED file. The intervals are bounded by 1 and length of the reference contig in cases where the interval extends beyond the length of the contig or is below 1.

For pair-end files, the start of the forward read to the end of the reverse read is designated the fragment interval. Fragment interval lengths that are smaller than the read lengths (meaning the reads are in Reverse-Forward orientation rather than Forward-Reverse) are discarded as ambiguous.

The following script was used to generate the BED file. It requires as input, the BAM file, a size file describing length of each reference contig, and average fragment size.

To get the size file for each reference contig, you can simply run `samtools faidx` on the genome FASTA file, then run `cut -f 1,2 genome.fasta.fai > size.file` to generate the size file.

For single-end files, use the distance predicted previously. For pair-end files, used 0 as the average fragment size to ignore extension. For pair-end data, this script can potentially use up a lot of memory.

```
import sys
from collections import defaultdict

fragSize = int(sys.argv[1])
refLengths = dict(x.strip().split() for x in open(sys.argv[2]))

if fragSize == 0:
    pairs = defaultdict(list)
    ref = ''
    for line in sys.stdin:
        meta = line.strip().split()

        if meta[2] != ref:
            ref = meta[2]
            pairs = defaultdict(list)

        pairs[meta[0]].append(meta)

        if len(pairs[meta[0]]) == 2:
            a = pairs[meta[0]][0]
            b = pairs[meta[0]][1]

            ref = a[2]
            refLength = int(refLengths[ref])

            aPos = int(a[3])
            aLength = len(a[9])
            aFlag = int(a[1])

            bPos = int(b[3])
            bLength = len(b[9])
            bFlag = int(b[1])

            if aFlag & 16:
                aPos += aLength
            else:
                bPos += bLength

            left = min(aPos,bPos)
            right = max(aPos,bPos)

            left = max(0,left)
            right = min(refLength,right)

            fragLength = right - left

            if fragLength > aLength and fragLength > bLength:
                print '\t'.join([ref,str(left),str(right),meta[0],str(fragLength)])

            del pairs[meta[0]]
else:
    for line in sys.stdin:
        meta = line.strip().split()
        flag = int(meta[1])
        ref = meta[2]
        pos = int(meta[3])
        length = len(meta[9])

        refLength = int(refLengths[ref])

        left = pos
        right = pos + fragSize

        if flag & 16:
            left = pos + length - fragSize
            right = pos + length

        left = max(left,0)
        right = min(refLength,right)

        print '\t'.join([ref,str(left), str(right),meta[0]])

#run this script by: samtools view lib.bam | python bam2bed.py fragLength sizes.file > lib.bed
```

#### Convert to "mid" format¶

Using the fragments .bed file, we also generate a "mid" file where the position of the fragment center is stored. Conceptually, the center of the fragment should be where the protein of interest is bound. In ChiP-seq experimentals, we are really only concerned with point-based data of the binding position. Extending the size of the fragment from the center just smooths out the binding density data.

```
import sys

inFile = open(sys.argv[1],'r')

for line in inFile:
        meta = line.strip().split('\t')
        ref,start,end = meta[:3]
        start = int(start)
        end = int(end)
        mid = start + ((end - start + 1) / 2)

        print ref + '\t' + str(mid)

#run this script by: python getMid.py lib.bed | sort -k 1,1 -k2,2n > lib.m
```

With this "mid" format, we can generate fragment bam/bed formatted data in whatever fragment length we want by using this script to generate a bed file with user defined fragment size:

```
import sys

inFile = open(sys.argv[1],'r')
chrSize = dict([x.strip().split() for x in open(sys.argv[2],'r').read().strip().split('\n')])
fragSize = int(sys.argv[3])

halfSize = fragSize / 2

count = 0
for line in inFile:
        ref, mid = line.strip().split()
        refLength = int(chrSize[ref])
        mid = int(mid)
        start = max(0,mid - halfSize)
        end = min(refLength,mid + halfSize)

        print '\t'.join(map(str,[ref,start,end,'frag' + str(count)]))
        count += 1

#use by: python mid2fragment.py lib.m chromosomeSizes fragmentSize > lib.fragmentSize.bed
#bed can then be optionally converted to bam with bedtools bed2bam command.
```

The resulting .bam file can be used with DeepTools to calculate normalization factors. The .bam/.bed file can be used to generate a bedgraph track using bedtools genomecov.

The resulting .bdg file can be converted to bigwig using UCSC's bedGraphToBigWig.

DeepTools' computeMatrix command can be used on multiple bigwig files and the annotations to extract coverage of region around features.

The following table shows the fragment metrics of each ChIP-seq library along with Sanchez's ChIP-seq libraries.

In [3]:

```
fragFile = open('data/frag.count')
smedCount = {}
dmelCount = {}
for line in fragFile:
    meta = line.strip().split()
    info = meta[1].split('.')
    if info[-2] == 'smed':
        smedCount[info[0]] = int(meta[0])
    elif info[-2] == 'dmel':
        dmelCount[info[0]] = int(meta[0])
fragFile.close()
```

In [4]:

```
metricTable = []
for lib, N_smed in smedCount.items():
    N_dmel = dmelCount[lib]
    info = lib.split('_')
    lab = 'Aboobaker'
    tissue = 'x1'
    marker = ''
    RNAi = ''
    rep = ''
    
    if info[0] == 'aa':
        marker = info[2].lower()
        if info[3] == 'na':
            RNAi = 'wt'
        else:
            RNAi = info[3]
        rep = info[4]
    elif info[0] == 'sa':
        lab = "Sanchez"
        tissue = info[1].lower()
        marker = info[2].lower()
        RNAi = info[3]
        rep = info[4]

    metricTable.append([lab,tissue,marker,RNAi,rep,N_smed,N_dmel])
        
metricTable.sort(key = lambda x : x[4])
metricTable.sort(key = lambda x : x[3])
metricTable.sort(key = lambda x : x[2])
metricTable.sort(key = lambda x : x[1])
metricTable.sort(key = lambda x : x[0])
metricTable.insert(0,['Lab','Sample','Histone Marker','RNAi','Replicate','# S.med reads','# D.mel reads'])
```

In [5]:

```
make_table(metricTable)
apply_theme('basic')
```

Out[5]:

|  |  |  |  |  |  |  |
| --- | --- | --- | --- | --- | --- | --- |
| **Lab** | **Sample** | **Histone&nbspMarker** | **RNAi** | **Replicate** | **#&nbspS.med&nbspreads** | **#&nbspD.mel&nbspreads** |
| Aboobaker | x1 | h3k27me3 | gfp | 01 | 10947961 | 1242189 |
| Aboobaker | x1 | h3k27me3 | gfp | 02 | 8235643 | 251940 |
| Aboobaker | x1 | h3k27me3 | gfp | 03 | 17618046 | 3688396 |
| Aboobaker | x1 | h3k27me3 | lpt | 01 | 9944787 | 841666 |
| Aboobaker | x1 | h3k27me3 | lpt | 02 | 8630637 | 366242 |
| Aboobaker | x1 | h3k27me3 | lpt | 03 | 15498068 | 3383720 |
| Aboobaker | x1 | h3k27me3 | wt | 01 | 50487823 | 6337 |
| Aboobaker | x1 | h3k4me1 | gfp | 01 | 15261263 | 1577088 |
| Aboobaker | x1 | h3k4me1 | gfp | 02 | 15682701 | 495370 |
| Aboobaker | x1 | h3k4me1 | gfp | 03 | 33322750 | 6639582 |
| Aboobaker | x1 | h3k4me1 | lpt | 01 | 10253234 | 1108472 |
| Aboobaker | x1 | h3k4me1 | lpt | 02 | 15597907 | 652922 |
| Aboobaker | x1 | h3k4me1 | lpt | 03 | 32760045 | 6375771 |
| Aboobaker | x1 | h3k4me1 | wt | 01 | 90880091 | 9900 |
| Aboobaker | x1 | h3k4me3 | gfp | 01 | 14724474 | 3572880 |
| Aboobaker | x1 | h3k4me3 | gfp | 02 | 13252896 | 993932 |
| Aboobaker | x1 | h3k4me3 | gfp | 03 | 26551125 | 8812331 |
| Aboobaker | x1 | h3k4me3 | lpt | 01 | 8932219 | 2507849 |
| Aboobaker | x1 | h3k4me3 | lpt | 02 | 11564615 | 1016715 |
| Aboobaker | x1 | h3k4me3 | lpt | 03 | 29595862 | 11239655 |
| Aboobaker | x1 | h3k4me3 | wt | 01 | 22977210 | 2319 |
| Aboobaker | x1 | input | gfp | 01 | 15170601 | 1507771 |
| Aboobaker | x1 | input | gfp | 02 | 13115084 | 394458 |
| Aboobaker | x1 | input | gfp | 03 | 21644170 | 3322263 |
| Aboobaker | x1 | input | lpt | 01 | 11723820 | 849039 |
| Aboobaker | x1 | input | lpt | 02 | 12748504 | 408706 |
| Aboobaker | x1 | input | lpt | 03 | 21706982 | 3484726 |
| Aboobaker | x1 | input | wt | 01 | 193998307 | 23024 |
| Sanchez | whole | h3k4me3 | mll | SRR2726657 | 18200382 | 7770373 |
| Sanchez | whole | h3k4me3 | mll | SRR2726658 | 17906141 | 8970148 |
| Sanchez | whole | h3k4me3 | na | SRR2726631 | 28010485 | 7361418 |
| Sanchez | whole | h3k4me3 | set | SRR2726633 | 18534571 | 7904641 |
| Sanchez | whole | h3k4me3 | set | SRR2726634 | 17664403 | 6613211 |
| Sanchez | whole | h3k4me3 | unc22 | SRR2726637 | 23109327 | 7396279 |
| Sanchez | whole | h3k4me3 | unc22 | SRR2726638 | 24407077 | 7856779 |
| Sanchez | whole | h3k4me3 | unc22 | SRR2726661 | 24138690 | 7844356 |
| Sanchez | whole | h3k4me3 | unc22 | SRR2726662 | 22388217 | 6789016 |
| Sanchez | whole | input | mll | SRR2726659 | 22172419 | 6023622 |
| Sanchez | whole | input | mll | SRR2726660 | 21981881 | 5935414 |
| Sanchez | whole | input | na | SRR2726632 | 30763052 | 7986374 |
| Sanchez | whole | input | set | SRR2726635 | 22387206 | 4239682 |
| Sanchez | whole | input | set | SRR2726636 | 23937089 | 4783740 |
| Sanchez | whole | input | unc22 | SRR2726639 | 27187779 | 5502076 |
| Sanchez | whole | input | unc22 | SRR2726640 | 21937325 | 4568731 |
| Sanchez | whole | input | unc22 | SRR2726663 | 24140173 | 4545750 |
| Sanchez | whole | input | unc22 | SRR2726664 | 25479059 | 5014488 |
| Sanchez | x1 | h3k4me3 | mll | SRR2726641 | 3698422 | 32733358 |
| Sanchez | x1 | h3k4me3 | mll | SRR2726642 | 2406578 | 21035203 |
| Sanchez | x1 | h3k4me3 | na | SRR2726607 | 765305 | 20051292 |
| Sanchez | x1 | h3k4me3 | na | SRR2726608 | 2370282 | 18811157 |
| Sanchez | x1 | h3k4me3 | set | SRR2726615 | 3089339 | 16214506 |
| Sanchez | x1 | h3k4me3 | set | SRR2726616 | 3432068 | 15474774 |
| Sanchez | x1 | h3k4me3 | unc22 | SRR2726623 | 3550931 | 15244101 |
| Sanchez | x1 | h3k4me3 | unc22 | SRR2726624 | 3847370 | 17332482 |
| Sanchez | x1 | h3k4me3 | unc22 | SRR2726649 | 5392596 | 30787848 |
| Sanchez | x1 | h3k4me3 | unc22 | SRR2726650 | 3578055 | 29897763 |
| Sanchez | x1 | input | mll | SRR2726643 | 4908991 | 37403613 |
| Sanchez | x1 | input | mll | SRR2726644 | 5314668 | 32665290 |
| Sanchez | x1 | input | na | SRR2726611 | 1065744 | 45095004 |
| Sanchez | x1 | input | na | SRR2726612 | 3828452 | 40596140 |
| Sanchez | x1 | input | set | SRR2726617 | 4916030 | 14608420 |
| Sanchez | x1 | input | set | SRR2726618 | 6047926 | 16536895 |
| Sanchez | x1 | input | unc22 | SRR2726625 | 4511192 | 12280642 |
| Sanchez | x1 | input | unc22 | SRR2726626 | 5054323 | 16291320 |
| Sanchez | x1 | input | unc22 | SRR2726651 | 4585702 | 26387945 |
| Sanchez | x1 | input | unc22 | SRR2726652 | 4891249 | 35638632 |
| Sanchez | xins | h3k4me3 | mll | SRR2726645 | 1455176 | 30232363 |
| Sanchez | xins | h3k4me3 | mll | SRR2726646 | 2006959 | 33406953 |
| Sanchez | xins | h3k4me3 | na | SRR2726609 | 164450 | 16881802 |
| Sanchez | xins | h3k4me3 | na | SRR2726610 | 829201 | 16904721 |
| Sanchez | xins | h3k4me3 | set | SRR2726619 | 2342780 | 19610250 |
| Sanchez | xins | h3k4me3 | set | SRR2726620 | 2956139 | 17816949 |
| Sanchez | xins | h3k4me3 | unc22 | SRR2726627 | 2189668 | 19294838 |
| Sanchez | xins | h3k4me3 | unc22 | SRR2726628 | 2407994 | 17196763 |
| Sanchez | xins | h3k4me3 | unc22 | SRR2726653 | 1529795 | 29508003 |
| Sanchez | xins | h3k4me3 | unc22 | SRR2726654 | 1697405 | 32121357 |
| Sanchez | xins | input | mll | SRR2726647 | 2661163 | 42277893 |
| Sanchez | xins | input | mll | SRR2726648 | 3360884 | 43768571 |
| Sanchez | xins | input | na | SRR2726613 | 435762 | 39014102 |
| Sanchez | xins | input | na | SRR2726614 | 2364649 | 42748588 |
| Sanchez | xins | input | set | SRR2726621 | 3829420 | 19445468 |
| Sanchez | xins | input | set | SRR2726622 | 3706717 | 15939505 |
| Sanchez | xins | input | unc22 | SRR2726629 | 3355890 | 18453252 |
| Sanchez | xins | input | unc22 | SRR2726630 | 3620972 | 17576637 |
| Sanchez | xins | input | unc22 | SRR2726655 | 2929435 | 43136208 |
| Sanchez | xins | input | unc22 | SRR2726656 | 2855532 | 39216193 |

In [110]:

```
%%HTML
<a id='sec03'></a>
```

## ChIP-seq normalization¶

There are two general types of normalization that can be performed on ChIP-seq data. A normalization to input to remove read mappability biases or sequencing biases. And a normalization across samples for comparison of regions among samples.

There doesn't seem to be a standard way of incorporating both normalization methods to generate one single output track. The strategy for producing the final signal track in this analysis is to subtract the input track from sample track after normalizing coverage across all samples and input.

#### Normalization to input¶

Normalization to input transforms the sample track in a way to account for biases from mapping, sequencing, and chemistry.

To obtain a comparable input track, the sample and input needs to be scaled correctly. Most chip-seq analysis scale sample to input by scaling according to library size. The optimal way would be to the scale by comparing the background in both sample and input. I am going to use the SES method described in this paper:

```
http://www.ncbi.nlm.nih.gov/pmc/articles/PMC3342857/
```

DeepTool's API was used to calculate the SES scaling factors:

```
from deeptools import SES_scaleFactor

    result = SES_scaleFactor.estimateScaleFactor(['samplePath','inputPath'],500,200000,1,numberOfProcessors=32)
    print str(result['size_factors_SES'][0]) + '\t' + str(result['size_factors_SES'][1])
```

One caveat about input samples. There is anectodal evidence from other bioinformaticians suggesting input libraries might be biased to also include the IP signal. Since input also goes through the cross-linking and sonication process, protein-bound fragments will tend not to be fragmented, potentially resulting in a signal for any protein-bound chromatin. The Sono-seq paper seems to also suggest this:

```
Mapping accessible chromatin regions using Sono-Seq (http://www.pnas.org/content/106/35/14926.short)
```

If the signal from input samples are inclusive of the IP signal, then normalization to input will potentially bury real signal.

#### Normalization across samples¶

Theory behind chip-seq normalization using spike-in samples can be found in this paper:

```
Bin Hu, et al. Biological chromodynamics: a general method for measuring protein occupancy across the genome by calibrating ChIP-seq. Nucl. Acids Res. (2015) doi: 10.1093/nar/gkv670.

http://nar.oxfordjournals.org/content/early/2015/06/30/nar.gkv670.full
```

Cells for each sample (GFP01, GFP02, LTP01, LPT02) were FACS sorted for X1 population and spiked-in with a proportional (3%) number of Drosophila S2 cells.

Each sequenced sample contains reads representing occupancy of histone marker on the S.med and D.mel genome:

- Reads smed = # reads for S.med
- Reads dmel = # reads for D.mel

For S.med and D.mel, the number of reads is a factor of the number of input cells, the histone occupancy rate, and technical residuals.

- N = number of cells
- O = occupancy rate
- R = residuals

- Reads = N \* O \* R

- Reads smed = N smed \* O smed \* R smed
- Reads dmel = N dmel \* O dmel \* R dmel

Occupancy is defined as the probability that a position on the genome is bound by the protein of interest. Occupancy rate is the average across all the positions on the genome.

For each sample, the proportion of S.med reads to D.mel reads can be written as:

- Reads smed / Reads dmel = (N smed  *O smed*  R smed) / (N dmel  *O dmel*  R dmel)

What we are interested in is the histone occupancy rate of S.med (O smed). The above equation can be rearranged to solve for O smed:

- O smed = (Reads smed  *N dmel*  O dmel  *R dmel) / (Reads dmel*  N smed \* R smed)

The histone occupancy rate of D.mel (O dmel) is a constant since we are spiking in the same S2 cells in all samples.

The residuals (R) include variances from sonication, IP, library prep, sequencing. Residuals do NOT include histone occupancy, which can be different among S.med samples.

While the residuals for each sample maybe different, the ratio of S.med residuals (R smed) to D.mel residuals (R dmel) can be assumed to be constant among all samples undergoing the same immunoprecipitation. Our four samples (GFP01, GFP02, LPT01, LPT02), under same immunoprecipitation should have a constant O dmel and constant R dmel / R smed.

- O dmel \* R dmel / R dmel = constant = Alpha

Substituting for Alpha in previous equation:

- O smed = ((Reads smed  *N dmel) / (Reads dmel*  N smed)) \* Alpha

Designate ((Reads smed  *N dmel) / (Reads dmel*  N smed)) as Scale Factor:

- Scale Factor = ((Reads smed  *N dmel) / (Reads dmel*  N smed))
- O smed = Scale Factor \* Alpha

The number of S.med and D.mel cells (N smed and N dmel) mixed in each sample can be obtained by the initial measurement of cell concentration after FACS and spike-in aliquot. Alternatively, we can estimate N smed and N dmel using S.med / D.mel read proportions of our input samples.

According to the paper, this scaling factor is then applied to the density distribution of the coverage (ie. Reads per million). Conversion of raw read coverage to a density distribution can be done by dividing each coverage value per base by the total number of reads, then multiplying by a million. The multiplication by a million make the number bigger and easier to work with.

When putting both RPM conversion and scaling factor together, we are essentially dividing each coverage value per base by:

- (1e6 / Reads smed)  *((Reads smed*  N dmel) / (Reads dmel \* N smed))

We can get rid of the 1e6 as a constant and take out Reads smed. We are left with:

- Scaling factor = N dmel / (Reads dmel \* N smed)

This scaling factor can then be used on the raw read coverage (bedgraph) to normalize samples.

#### Generate comparable coverage tracks among all samples and inputs¶

We can calculate a scaling for for each sample and its respective input using the SES method. We can also calculate a scaling factor among samples using the spike-in method. To consolidate these two scaling factors, we can simply scale all samples according to the spike-in normalization and then scale the respective inputs according to the SES factor while setting the sample to 1.0.

For example:

```
SES scaling factor
h3k4me3 gfp01 : input gfp01 = 1   : 0.6
h3k4me3 gfp02 : input gfp02 = 1   : 0.4
h3k4me3 lpt01 : input lpt01 = 1   : 0.7
h3k4me3 lpt02 : input lpt02 = 0.8 : 1 or 1 : 1.25

Spike-in scaling factor
h3k4me3 gfp01    1
h3k4me3 gfp02    0.6
h3k4me3 lpt01    0.9
h3k4me3 lpt02    0.7

Final scaling factor
h3k4me3 gfp01    1
h3k4me3 gfp02    0.6
h3k4me3 lpt01    0.9
h3k4me3 lpt02    0.7
input gfp01    0.6   (1 * 0.6)
input gfp02    0.24  (0.6 * 0.4)
input lpt01    0.63  (0.9 * 0.7)
input lpt02    0.875 (1.25 * 0.7)
```

This final scaling will produce coverage tracks that are comparable among the same IPs.

The following are normalization factors calculated for ChIP-seq libraries used in the analysis.

In [11]:

```
def scaleFactor(sample, inp):
    R_smed, R_dmel = sample
    N_smed, N_dmel = inp
    
    ScF = N_dmel / (float(R_dmel) * N_smed)
    
    return ScF

def scaleFactorRPM(sample, inp):
    R_smed, R_dmel = sample
    N_smed, N_dmel = inp
    
    ScF = ((float(R_smed) * N_dmel) / (float(R_dmel) * N_smed)) * (1e6 / R_smed)
    
    return ScF
```

In [12]:

```
scf = {}
for sampleLib, sampleCount in smedCount.items():
    meta = sampleLib.split('_')
    if meta[0] == 'aa' and meta[3] != 'na':
        meta[2] = 'input'
        inputLib = '_'.join(meta)

        inputCount = smedCount[inputLib]

        dmelSampleCount = dmelCount[sampleLib]
        dmelInputCount = dmelCount[inputLib]

        f = scaleFactor([sampleCount,dmelSampleCount],[inputCount,dmelInputCount])
        scf[sampleLib] = f
```

In [13]:

```
sesFile = open('data/aa.ses.factors')
ses = {}
for line in sesFile:
    meta = line.strip().split()
    sample = meta[0].split('/')[-1].split('.')[0]
    s_factor = float(meta[2])
    i_factor = float(meta[3])
    if s_factor < 1:
        i_factor = 1 / s_factor
        s_factor = 1.0
    ses[sample] = i_factor
    
sesFile.close()
```

In [15]:

```
def generateFactorTable(mark):
    cmds = ''
    markTable = []
    libs = [[k,d] for k,d in scf.items() if k.split('_')[2] == mark]
    libs.sort(key = lambda x : x[0])
    m = max([x[1] for x in libs])
    
    norms = [[k,d / m] for k,d in libs]
    markTable.append(['Sample','Sample scaling factor','Input scaling factor'])
    samp = []
    inp = []
    for k,d in norms:    
        markTable.append([k, d, d * ses[k]])
        cmds += 'cat bdg/' + k + '.smed.100bp.bdg | python scale.py ' + \
        str(d) + ' > scaled/' + k + '.sample.bdg\n'
        cmds += 'cat bdg/' + k.replace(mark,'input') + '.smed.100bp.bdg | python scale.py ' + \
        str(d * ses[k]) + ' > scaled/' + k + '.input.bdg\n'
        
        samp.append(d)
        inp.append(d * ses[k])

    #print cmds
    return markTable

make_table(generateFactorTable('h3k4me3'))
apply_theme('basic')
```

Out[15]:

|  |  |  |
| --- | --- | --- |
| **Sample** | **Sample&nbspscaling&nbspfactor** | **Input&nbspscaling&nbspfactor** |
| aa\_x1\_h3k4me3\_gfp\_01 | 0.8822 | 0.6009 |
| aa\_x1\_h3k4me3\_gfp\_02 | 0.9597 | 0.6741 |
| aa\_x1\_h3k4me3\_gfp\_03 | 0.5524 | 0.5587 |
| aa\_x1\_h3k4me3\_lpt\_01 | 0.9158 | 0.3816 |
| aa\_x1\_h3k4me3\_lpt\_02 | 1.0000 | 0.6140 |
| aa\_x1\_h3k4me3\_lpt\_03 | 0.4530 | 0.4452 |

In [16]:

```
make_table(generateFactorTable('h3k4me1'))
apply_theme('basic')
```

Out[16]:

|  |  |  |
| --- | --- | --- |
| **Sample** | **Sample&nbspscaling&nbspfactor** | **Input&nbspscaling&nbspfactor** |
| aa\_x1\_h3k4me1\_gfp\_01 | 0.9646 | 0.9511 |
| aa\_x1\_h3k4me1\_gfp\_02 | 0.9293 | 1.0892 |
| aa\_x1\_h3k4me1\_gfp\_03 | 0.3538 | 0.5388 |
| aa\_x1\_h3k4me1\_lpt\_01 | 1.0000 | 0.6308 |
| aa\_x1\_h3k4me1\_lpt\_02 | 0.7515 | 0.8906 |
| aa\_x1\_h3k4me1\_lpt\_03 | 0.3854 | 0.5660 |

In [17]:

```
make_table(generateFactorTable('h3k27me3'))
apply_theme('basic')
```

Out[17]:

|  |  |  |
| --- | --- | --- |
| **Sample** | **Sample&nbspscaling&nbspfactor** | **Input&nbspscaling&nbspfactor** |
| aa\_x1\_h3k27me3\_gfp\_01 | 0.6702 | 0.4747 |
| aa\_x1\_h3k27me3\_gfp\_02 | 1.0000 | 0.6145 |
| aa\_x1\_h3k27me3\_gfp\_03 | 0.3486 | 0.2810 |
| aa\_x1\_h3k27me3\_lpt\_01 | 0.7208 | 0.6031 |
| aa\_x1\_h3k27me3\_lpt\_02 | 0.7332 | 0.4882 |
| aa\_x1\_h3k27me3\_lpt\_03 | 0.3974 | 0.2628 |

In [111]:

```
%%HTML
<a id='sec04'></a>
```

## ChIP-seq analysis¶

DeepTools was used to generate a data matrix of profiles based on annotated loci. This matrix file was parsed into data file consisting of coverages for each sample/IP type. This data file represents coverage at each 50bp bin across 5kb region of each loci (2.5k up and downstream of each loci start position).

In [25]:

```
import math
def smooth(x,window_len=5,window='hanning'):
    if window_len<3:
        return x

    if not window in ['flat', 'hanning', 'hamming', 'bartlett', 'blackman']:
        raise ValueError, "Window is on of 'flat', 'hanning', 'hamming', 'bartlett', 'blackman'"

    s=np.r_[x[window_len-1:0:-1],x,x[-1:-window_len:-1]]
    if window == 'flat':
        w=np.ones(window_len,'d')
    else:
        w=eval('np.'+window+'(window_len)')

    y=np.convolve(w/w.sum(),s,mode='valid')
    return y

def getFC(control,treatment,maxThresh):
    if control == 0 and treatment == 0:
        return 0
    elif control == 0 and treatment > 0:
        return maxThresh
    elif control > 0 and treatment == 0:
        return -maxThresh
    else:
        return min(maxThresh,max(-maxThresh,math.log(treatment / control,2)))
    
data_gfp = defaultdict(lambda : defaultdict())
data_lpt = defaultdict(lambda : defaultdict())
data_delta = defaultdict(lambda : defaultdict())
data_count = defaultdict(lambda : defaultdict())
data_fc = defaultdict(lambda : defaultdict())

df_data = []

dataFile = open('data/aa.data')
lids = []
for line in dataFile:
    lid,mark,gfp,lpt,delta,count = line.strip().split()
    lids.append(lid)
    gfp = smooth(map(float,gfp.split(',')),10)
    lpt = smooth(map(float,lpt.split(',')),10)
    delta = lpt - gfp
    
    signal_max = max(gfp.max(),lpt.max())
    gfp_norm = [0.0] * 109
    lpt_norm = [0.0] * 109
    delta_norm = [0.0] * 109
    fc = [0.0] * 109
    if signal_max > 0:
        gfp_norm = np.array([x / signal_max for x in gfp])
        lpt_norm = np.array([x / signal_max for x in lpt])
        delta_norm = lpt_norm - gfp_norm
        fc = [getFC(gfp_norm[i],lpt_norm[i],5) if gfp_norm[i] != lpt_norm[i] \
                                  else 0 for i in range(len(gfp_norm))]
    
    count = map(float,count.split(','))
    count_max = max(count)
    count_min = min(count)
    count_sum = sum(count)
    count_mean = count_sum / float(len(count))
    
    df_data.append([mark,gfp,lpt,delta,gfp_norm,lpt_norm,delta_norm,fc,count_max,count_min,count_sum,count_mean])

dataFile.close()
df_data = pd.DataFrame(df_data,\
                       columns=['mark','gfp','lpt','delta','gfp_norm',\
                                'lpt_norm','delta_norm','fc','count_max','count_min',\
                                'count_sum','count_mean'],
                       index = lids)
```

#### Outlier identification and removal¶

The following shows the distribution of max coverages from -500 to 800 around the TSS for all loci.

We manually defined the outlier threshold to be:

- 80 for h3k4me3
- 10 for h3k4me1
- 10 for h3k27me3

In [31]:

```
def maxRegion(x):
    #-500 to +800 around tss
    return max(x[44:73])

h3k4me3_gfp_regionMag = pd.Series(df_data[df_data['mark'] == 'h3k4me3']['gfp'].apply(np.max))
h3k4me1_gfp_regionMag = pd.Series(df_data[df_data['mark'] == 'h3k4me1']['gfp'].apply(np.max))
h3k27me3_gfp_regionMag = pd.Series(df_data[df_data['mark'] == 'h3k27me3']['gfp'].apply(np.max))

h3k4me3_lpt_regionMag = pd.Series(df_data[df_data['mark'] == 'h3k4me3']['lpt'].apply(np.max))
h3k4me1_lpt_regionMag = pd.Series(df_data[df_data['mark'] == 'h3k4me1']['lpt'].apply(np.max))
h3k27me3_lpt_regionMag = pd.Series(df_data[df_data['mark'] == 'h3k27me3']['lpt'].apply(np.max))

fig,ax = plt.subplots(3,figsize=[10,5])
h3k4me3_gfp_regionMag[h3k4me3_gfp_regionMag < 100].hist(bins=100,ax=ax[0],alpha=1)
h3k4me1_gfp_regionMag[h3k4me1_gfp_regionMag < 50].hist(bins=100,ax=ax[1],alpha=1)
h3k27me3_gfp_regionMag[h3k27me3_gfp_regionMag < 50].hist(bins=100,ax=ax[2],alpha=1)

ax[0].set_title('h3k4me3',fontsize=15)
ax[0].set_xlim(0,100)
ax[1].set_title('h3k4me1',fontsize=15)
ax[1].set_xlim(0,50)
ax[2].set_title('h3k27me3',fontsize=15)
ax[2].set_xlim(0,50)
fig.tight_layout()
fig.suptitle('distribution of magnitudes',y=1.05,fontsize=18)
fig.tight_layout()
```

In [29]:

```
h3k4me3_magOutlier = set(h3k4me3_gfp_regionMag[h3k4me3_gfp_regionMag > 80].index) \
| set(h3k4me3_lpt_regionMag[h3k4me3_lpt_regionMag > 80].index)
h3k4me1_magOutlier = set(h3k4me3_gfp_regionMag[h3k4me1_gfp_regionMag > 20].index) \
| set(h3k4me3_lpt_regionMag[h3k4me1_lpt_regionMag > 20].index)
h3k27me3_magOutlier = set(h3k4me3_gfp_regionMag[h3k27me3_gfp_regionMag > 10].index) \
| set(h3k4me3_lpt_regionMag[h3k27me3_lpt_regionMag > 10].index)
mark_outlier = h3k4me3_magOutlier | h3k4me1_magOutlier | h3k27me3_magOutlier
```

In [30]:

```
print 'number of outliers'
print 'h3k4me3:', len(h3k4me3_magOutlier)
print 'h3k4me1:', len(h3k4me1_magOutlier)
print 'h3k27me3:', len(h3k27me3_magOutlier)
print 'union of all three marks:', len(mark_outlier)
```

```
number of outliers
h3k4me3: 352
h3k4me1: 406
h3k27me3: 298
union of all three marks: 777
```

#### ChIP-seq profiles¶

The following shows the ChIP-seq profile for h3k4me3, h3k4me1, h3k27me3 histone marks in GFP and smed-LPT RNAi X1 cells. Each colored profile line represent an averaged profile of loci categorized by FACS. For this first figure, dark blue represents X1 category, Light blue represents X1 category, and orange represents Xins category.

In [82]:

```
def plotProfile(mark,gids,signal,count_thresh,c,ax):
    window = int(math.ceil(5000.0 / 109))
    xticks = range(-2500,2500,window)
    
    sns.tsplot(list(df_data[(df_data['mark'] == mark) & (df_data['count_max'] >= count_thresh)].ix[gids].dropna()[signal]),\
                   ax=ax,color=c,time=xticks)
    
def plotProfileGroups(groups,leg,signal='_norm',colors=['#1C637A','#81CCE6','#FA8D20','#4AB545']):
    fig, ax = plt.subplots(3,3,figsize=[15,8])
    
    for i,group in enumerate(groups):
        plotProfile('h3k4me3',group,'gfp' + signal,10,colors[i],ax[0][0])
        plotProfile('h3k4me3',group,'lpt' + signal,10,colors[i],ax[0][1])
        plotProfile('h3k4me3',group,'fc',10,colors[i],ax[0][2])
   
    for i,group in enumerate(groups):
        plotProfile('h3k4me1',group,'gfp' + signal,10,colors[i],ax[1][0])
        plotProfile('h3k4me1',group,'lpt' + signal,10,colors[i],ax[1][1])
        plotProfile('h3k4me1',group,'fc',10,colors[i],ax[1][2])
   
    for i,group in enumerate(groups):
        plotProfile('h3k27me3',group,'gfp' + signal,5,colors[i],ax[2][0])
        plotProfile('h3k27me3',group,'lpt' + signal,5,colors[i],ax[2][1])
        plotProfile('h3k27me3',group,'fc',5,colors[i],ax[2][2])
       
    ax[0][0].set_title('GFP RNAi',fontsize=20)
    ax[0][1].set_title('LPT RNAi',fontsize=20)
    ax[0][2].set_title('Log2 fold-change',fontsize=20)
    ax[0][0].set_ylabel('h3k4me3',fontsize=20)
    ax[1][0].set_ylabel('h3k4me1',fontsize=20)
    ax[2][0].set_ylabel('h3k27me3',fontsize=20)
    
    def setLim(ax):
        ymin = min(ax[0].get_ylim()[0],ax[1].get_ylim()[0])
        ymax = max(ax[0].get_ylim()[1],ax[1].get_ylim()[1])
        m = max(ax[0].get_ylim()[1],ax[1].get_ylim()[1])
        ax[0].set_ylim(ymin,ymax)
        ax[1].set_ylim(ymin,ymax)
        
    ax[0][2].set_ylim(-0.6,0.6)
    ax[1][2].set_ylim(-0.6,0.6)
    ax[2][2].set_ylim(-0.6,0.6)
        
    setLim(ax[0])
    setLim(ax[1])
    setLim(ax[2])
```

In [85]:

```
plotProfileGroups([x1_lids - mark_outlier, x2_lids - mark_outlier, xins_lids - mark_outlier]\
                  ,['X1','X2','Xins'],signal='')
```

In the following figure, dark blue represents X1/X2 category, Light blue represents X2/Xins category, and orange represents X1/Xins category.

In [86]:

```
plotProfileGroups([x12_lids - mark_outlier, x2ins_lids - mark_outlier, x1ins_lids - mark_outlier]\
                  ,['X1/X2','X2/Xins','X1/Xins'],signal='')
```

In [98]:

```
def getCorr(mark,group,fc):
    df = pd.DataFrame(df_data[df_data['mark'] == mark].ix[group].dropna()['delta_norm']).join(lpt_fc,how='inner')
    df = df[df['abs_fc'] >= fc]
    l = len(df['delta_norm'][0])
    c = [ss.spearmanr([x[i] for x in df['delta_norm']],df['fc']) for i in range(l)]

    return c

def plotCorr(mark,lids,ax,fc):
    c = getCorr(mark,lids,fc)
    window = int(math.ceil(5000.0 / 109))
    idx = range(-2500,2500,window)

    c = pd.DataFrame(c,columns=['correlation','pval'])
    nonsig = c.copy()
    nonsig[nonsig['pval'] < 0.001] = np.nan
    sig = c.copy()
    sig[sig['pval'] > 0.001] = np.nan

    pd.Series(list(c['correlation']),index=idx)[32:78].plot(ax=ax)
    pd.Series(list(nonsig['correlation']),index=idx)[32:78].plot(ax=ax,c='white',alpha=0.7)
```

#### Correlation¶

The following shows correlation between the RNA-seq fold-change of loci down/up-regulated after smed-LPT RNAi and the respective h3k4me3, h3k4me1 and h3k27me3 histone marks profiles. The correlation profiles represents a region from -1000 to +1000 of the TSS.

In [99]:

```
fig,ax = plt.subplots(3,3,figsize=[10,6])

plotCorr('h3k4me3',x1_lids,ax[0][0],math.log(1,2))
plotCorr('h3k4me3',x2_lids,ax[0][1],math.log(1,2))
plotCorr('h3k4me3',xins_lids,ax[0][2],math.log(1,2))

plotCorr('h3k4me1',x1_lids,ax[1][0],math.log(1,2))
plotCorr('h3k4me1',x2_lids,ax[1][1],math.log(1,2))
plotCorr('h3k4me1',xins_lids,ax[1][2],math.log(1,2))

plotCorr('h3k27me3',x1_lids,ax[2][0],math.log(1,2))
plotCorr('h3k27me3',x2_lids,ax[2][1],math.log(1,2))
plotCorr('h3k27me3',xins_lids,ax[2][2],math.log(1,2))

plotCorr('h3k4me3',x1_lids,ax[0][0],math.log(2,2))
plotCorr('h3k4me3',x2_lids,ax[0][1],math.log(2,2))
plotCorr('h3k4me3',xins_lids,ax[0][2],math.log(2,2))

plotCorr('h3k4me1',x1_lids,ax[1][0],math.log(2,2))
plotCorr('h3k4me1',x2_lids,ax[1][1],math.log(2,2))
plotCorr('h3k4me1',xins_lids,ax[1][2],math.log(2,2))

plotCorr('h3k27me3',x1_lids,ax[2][0],math.log(2,2))
plotCorr('h3k27me3',x2_lids,ax[2][1],math.log(2,2))
plotCorr('h3k27me3',xins_lids,ax[2][2],math.log(2,2))

ax[0][0].set_title('X1',fontsize=18)
ax[0][1].set_title('X2',fontsize=18)
ax[0][2].set_title('Xins',fontsize=18)

ax[0][0].set_ylabel('h3k4me3',fontsize=20)
ax[1][0].set_ylabel('h3k4me1',fontsize=20)
ax[2][0].set_ylabel('h3k27me3',fontsize=20)

for row in ax:
    for col in row:
        col.set_ylim(-0.5,0.5)
        col.axhline(y=0,xmin=0,xmax=1,color='k',linewidth=1)

fig.tight_layout()
```

In [102]:

```
fig,ax = plt.subplots(3,3,figsize=[10,6])

plotCorr('h3k4me3',x12_lids,ax[0][0],math.log(1,2))
plotCorr('h3k4me3',x2ins_lids,ax[0][1],math.log(1,2))
plotCorr('h3k4me3',x0_lids,ax[0][2],math.log(1,2))

plotCorr('h3k4me1',x12_lids,ax[1][0],math.log(1,2))
plotCorr('h3k4me1',x2ins_lids,ax[1][1],math.log(1,2))
plotCorr('h3k4me1',x0_lids,ax[1][2],math.log(1,2))

plotCorr('h3k27me3',x12_lids,ax[2][0],math.log(1,2))
plotCorr('h3k27me3',x2ins_lids,ax[2][1],math.log(1,2))
plotCorr('h3k27me3',x0_lids,ax[2][2],math.log(1,2))

plotCorr('h3k4me3',x12_lids,ax[0][0],math.log(2,2))
plotCorr('h3k4me3',x2ins_lids,ax[0][1],math.log(2,2))
plotCorr('h3k4me3',x0_lids,ax[0][2],math.log(2,2))

plotCorr('h3k4me1',x12_lids,ax[1][0],math.log(2,2))
plotCorr('h3k4me1',x2ins_lids,ax[1][1],math.log(2,2))
plotCorr('h3k4me1',x0_lids,ax[1][2],math.log(2,2))

plotCorr('h3k27me3',x12_lids,ax[2][0],math.log(2,2))
plotCorr('h3k27me3',x2ins_lids,ax[2][1],math.log(2,2))
plotCorr('h3k27me3',x0_lids,ax[2][2],math.log(2,2))

ax[0][0].set_title('X1/X2',fontsize=18)
ax[0][1].set_title('X2/Xins',fontsize=18)
ax[0][2].set_title('Unenriched',fontsize=18)

ax[0][0].set_ylabel('h3k4me3',fontsize=20)
ax[1][0].set_ylabel('h3k4me1',fontsize=20)
ax[2][0].set_ylabel('h3k27me3',fontsize=20)

for row in ax:
    for col in row:
        col.set_ylim(-0.5,0.5)
        col.axhline(y=0,xmin=0,xmax=1,color='k',linewidth=1)

fig.tight_layout()
```

In [112]:

```
%%HTML
<a id='sec05'></a>
```

## Appendix¶

#### Data sources used¶

The following RNA-seq libraries were used. The first column is the Run accession ID. The second column is the renamed file in the format of:

```
"library type, lab, sample, antibody, RNAi, time-point, Run ID", delimited by underscore.


SRR2726607    chip_sanchez_x1_H3K4me3_na_na_SRR2726607
SRR2726608    chip_sanchez_x1_H3K4me3_na_na_SRR2726608
SRR2726609    chip_sanchez_xins_H3K4me3_na_na_SRR2726609
SRR2726610    chip_sanchez_xins_H3K4me3_na_na_SRR2726610
SRR2726611    chip_sanchez_x1_Input_na_na_SRR2726611
SRR2726612    chip_sanchez_x1_Input_na_na_SRR2726612
SRR2726613    chip_sanchez_xins_Input_na_na_SRR2726613
SRR2726614    chip_sanchez_xins_Input_na_na_SRR2726614
SRR2726615    chip_sanchez_x1_H3K4me3_set_na_SRR2726615
SRR2726616    chip_sanchez_x1_H3K4me3_set_na_SRR2726616
SRR2726617    chip_sanchez_x1_Input_set_na_SRR2726617
SRR2726618    chip_sanchez_x1_Input_set_na_SRR2726618
SRR2726619    chip_sanchez_xins_H3K4me3_set_na_SRR2726619
SRR2726620    chip_sanchez_xins_H3K4me3_set_na_SRR2726620
SRR2726621    chip_sanchez_xins_Input_set_na_SRR2726621
SRR2726622    chip_sanchez_xins_Input_set_na_SRR2726622
SRR2726623    chip_sanchez_x1_H3K4me3_unc22_na_SRR2726623
SRR2726624    chip_sanchez_x1_H3K4me3_unc22_na_SRR2726624
SRR2726625    chip_sanchez_x1_Input_unc22_na_SRR2726625
SRR2726626    chip_sanchez_x1_Input_unc22_na_SRR2726626
SRR2726627    chip_sanchez_xins_H3K4me3_unc22_na_SRR2726627
SRR2726628    chip_sanchez_xins_H3K4me3_unc22_na_SRR2726628
SRR2726629    chip_sanchez_xins_Input_unc22_na_SRR2726629
SRR2726630    chip_sanchez_xins_Input_unc22_na_SRR2726630
SRR2726631    chip_sanchez_whole_H3K4me3_na_na_SRR2726631
SRR2726632    chip_sanchez_whole_Input_na_na_SRR2726632
SRR2726633    chip_sanchez_whole_H3K4me3_set_na_SRR2726633
SRR2726634    chip_sanchez_whole_H3K4me3_set_na_SRR2726634
SRR2726635    chip_sanchez_whole_Input_set_na_SRR2726635
SRR2726636    chip_sanchez_whole_Input_set_na_SRR2726636
SRR2726637    chip_sanchez_whole_H3K4me3_unc22_na_SRR2726637
SRR2726638    chip_sanchez_whole_H3K4me3_unc22_na_SRR2726638
SRR2726639    chip_sanchez_whole_Input_unc22_na_SRR2726639
SRR2726640    chip_sanchez_whole_Input_unc22_na_SRR2726640
SRR2726641    chip_sanchez_x1_H3K4me3_mll_na_SRR2726641
SRR2726642    chip_sanchez_x1_H3K4me3_mll_na_SRR2726642
SRR2726643    chip_sanchez_x1_Input_mll_na_SRR2726643
SRR2726644    chip_sanchez_x1_Input_mll_na_SRR2726644
SRR2726645    chip_sanchez_xins_H3K4me3_mll_na_SRR2726645
SRR2726646    chip_sanchez_xins_H3K4me3_mll_na_SRR2726646
SRR2726647    chip_sanchez_xins_Input_mll_na_SRR2726647
SRR2726648    chip_sanchez_xins_Input_mll_na_SRR2726648
SRR2726649    chip_sanchez_x1_H3K4me3_unc22_na_SRR2726649
SRR2726650    chip_sanchez_x1_H3K4me3_unc22_na_SRR2726650
SRR2726651    chip_sanchez_x1_Input_unc22_na_SRR2726651
SRR2726652    chip_sanchez_x1_Input_unc22_na_SRR2726652
SRR2726653    chip_sanchez_xins_H3K4me3_unc22_na_SRR2726653
SRR2726654    chip_sanchez_xins_H3K4me3_unc22_na_SRR2726654
SRR2726655    chip_sanchez_xins_Input_unc22_na_SRR2726655
SRR2726656    chip_sanchez_xins_Input_unc22_na_SRR2726656
SRR2726657    chip_sanchez_whole_H3K4me3_mll_na_SRR2726657
SRR2726658    chip_sanchez_whole_H3K4me3_mll_na_SRR2726658
SRR2726659    chip_sanchez_whole_Input_mll_na_SRR2726659
SRR2726660    chip_sanchez_whole_Input_mll_na_SRR2726660
SRR2726661    chip_sanchez_whole_H3K4me3_unc22_na_SRR2726661
SRR2726662    chip_sanchez_whole_H3K4me3_unc22_na_SRR2726662
SRR2726663    chip_sanchez_whole_Input_unc22_na_SRR2726663
SRR2726664    chip_sanchez_whole_Input_unc22_na_SRR2726664
```

#### Software used¶

In [113]:

```
%%HTML
<a id='sec06'></a>
```

In [105]:

```
make_table([x.split('\t') for x in '''Software	Version
Kallisto	0.42
Sleuth	0.28.1
Bedtools	2.20.1
Picard tools	1.115
Samtools	1.2
HISAT2	2.0.4
StringTie	1.3.0
DeepTools	2.2.4
PASA	2.0.2
TransDecoder	3.0.0
GMAP	12.31.2015
Cap3	8.6.2013
Trimmomatic	0.32'''.split('\n')])
apply_theme('basic')
```

Out[105]:

|  |  |
| --- | --- |
| **Software** | **Version** |
| Kallisto | 0.42 |
| Sleuth | 0.28.1 |
| Bedtools | 2.20.1 |
| Picard&nbsptools | 1.115 |
| Samtools | 1.2 |
| HISAT2 | 2.0.4 |
| StringTie | 1.3.0 |
| DeepTools | 2.2.4 |
| PASA | 2.0.2 |
| TransDecoder | 3.0.0 |
| GMAP | 12.31.2015 |
| Cap3 | 8.6.2013 |
| Trimmomatic | 0.32 |
